# USP28 enables oncogenic transformation of respiratory cells, and its inhibition potentiates molecular therapy targeting mutant EGFR, BRAF and PI3K

**DOI:** 10.1002/1878-0261.13217

**Published:** 2022-04-30

**Authors:** Cristian Prieto‐Garcia, Oliver Hartmann, Michaela Reissland, Fabian Braun, Süleyman Bozkurt, Nikolett Pahor, Carmina Fuss, Andreas Schirbel, Christina Schülein‐Völk, Alexander Buchberger, Marco A. Calzado Canale, Mathias Rosenfeldt, Ivan Dikic, Christian Münch, Markus E. Diefenbacher

**Affiliations:** ^1^ Protein Stability and Cancer Group Department of Biochemistry and Molecular Biology University of Wuerzburg Germany; ^2^ Mildred Scheel Early Career Center Wuerzburg Germany; ^3^ Molecular Signaling Group Institute of Biochemistry II Goethe University Frankfurt Germany; ^4^ Protein Quality Control Institute of Biochemistry II Goethe University Frankfurt Germany; ^5^ Division of Endocrinology and Diabetes Department of Internal Medicine I University Hospital University of Wuerzburg Germany; ^6^ Department of Nuclear Medicine University Hospital University of Wuerzburg Germany; ^7^ Core Unit High‐Content Microscopy Biocenter University of Wuerzburg Germany; ^8^ Department of Biochemistry Biocenter University of Wuerzburg Germany; ^9^ Instituto Maimónides de Investigación Biomédica de Córdoba (IMIBIC) Spain; ^10^ Departamento de Biología Celular, Fisiología e Inmunología Universidad de Córdoba Spain; ^11^ Hospital Universitario Reina Sofía Córdoba Spain; ^12^ Institut für Pathologie Universitaetsklinikum Wuerzburg Germany; ^13^ Buchmann Institute for Molecular Life Sciences Goethe University Frankfurt Germany

**Keywords:** buparlisib, c‐MYC, gefitinib, lung cancer, USP28, vemurafenib

## Abstract

Oncogenic transformation of lung epithelial cells is a multistep process, frequently starting with the inactivation of tumour suppressors and subsequent development of activating mutations in proto‐oncogenes, such as members of the PI3K or MAPK families. Cells undergoing transformation have to adjust to changes, including altered metabolic requirements. This is achieved, in part, by modulating the protein abundance of transcription factors. Here, we report that the ubiquitin carboxyl‐terminal hydrolase 28 (USP28) enables oncogenic reprogramming by regulating the protein abundance of proto‐oncogenes such as c‐JUN, c‐MYC, NOTCH and ∆NP63 at early stages of malignant transformation. USP28 levels are increased in cancer compared with in normal cells due to a feed‐forward loop, driven by increased amounts of oncogenic transcription factors such as c‐MYC and c‐JUN. Irrespective of oncogenic driver, interference with USP28 abundance or activity suppresses growth and survival of transformed lung cells. Furthermore, inhibition of USP28 via a small‐molecule inhibitor resets the proteome of transformed cells towards a ‘premalignant’ state, and its inhibition synergizes with clinically established compounds used to target EGFR^L858R^‐, BRAF^V600E^‐ or PI3K^H1047R^‐driven tumour cells. Targeting USP28 protein abundance at an early stage via inhibition of its activity is therefore a feasible strategy for the treatment of early‐stage lung tumours, and the observed synergism with current standard‐of‐care inhibitors holds the potential for improved targeting of established tumours.

## Graphical abstract

In this study, we identified that USP28 enables oncogenic reprograming of respiratory cells and is required to permit transformation, irrespective of driver mutation. Furthermore, the inhibition of USP28 synergizes with targeted molecular therapy, demonstrating that targeting USP28 already at an early stage therefore is a promising strategy for the treatment of lung tumours in combination with personalized targeted therapy.

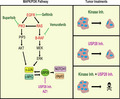

**Abbreviations**
AAV: adeno‐associated virus
ADC: adenocarcinoma
AKT2: RAC‐beta serine/threonine‐protein kinase 2
AT2: alveolar type II
BADJ: bronchoalveolar duct junction
BRAF: serine/threonine‐protein kinase B‐Raf
BUP: buparlisib
CC10: club cell protein
CRISPR: clustered regularly interspaced short palindromic repeat
Ctrl: control
DIF: differentiated
DUB: deubiquitinating enzyme
EGFR: epidermal growth factor receptor
GEF: gefitinib
H&E: haematoxylin and eosin
IF: immunofluorescence
IHC: immunohistochemistry
KP: Kras:Trp53
KPU: Kras:Trp53:Usp28
NSCLC: non‐small‐cell lung carcinoma
ONC: oncogenic
OS: overall survival
PFS: progression‐free survival
PIK3CA: phosphatidylinositol‐4,5‐bisphosphate 3‐kinase catalytic subunit alpha
PPS: postprogression survival
RAS: rat sarcoma virus
SCC: squamous cell carcinoma
sgRNA: single‐guide RNA
SPC: surfactant protein C
UD: undifferentiated
UPS: ubiquitin–proteasome system
USP: ubiquitin‐specific‐processing protease
VEM: vemurafenib
WT: wild‐type

## 1 Introduction

In the past decade, with the advent of targeted therapy, great advancements towards the treatment of progressed non‐small‐cell lung cancer (NSCLC) in distinct patient cohorts were achieved [1], while patients with early disease do not benefit from these new treatments [2, 3]. For this cohort, the curative treatment, still today, is the surgical resection of a lung lobe. This is a severe procedure, inflicting major damage, requires an extended recovery time and can result in therapy‐induced mortality [4, 5]. Furthermore, therapy failure in late‐stage tumours by establishment of treatment escape mechanisms is a common observation in NSCLC, significantly affecting patient survival [6, 7]. Overall, survival rates have only marginally improved and most patients still succumb to the disease [1].

Therefore, targeting of common essential pathways and exploiting tumour intrinsic vulnerabilities hold the potential to improve current treatment not only for late‐stage but also for early‐stage patients. One central cellular component tumour cells alter during oncogenic transformation is the ubiquitin–proteasome system (UPS) [8, 9]. The dysregulation of the UPS is a prerequisite for tumour cells to tolerate increased proliferation, metabolic changes, immune evasion and proteostatic stress management [10]. All these processes are ‘hallmarks of cancer’ and therefore significantly contribute to disease progression, therapy failure and shorted survival. Therefore, cancer cells, when compared to nontransformed cells, are dependent on the ubiquitin system [11, 12]. As a consequence, tumour cells develop exploitable dependencies towards the expression and abundance of discreet members of the UPS.

Despite the prominent involvement of the UPS in cancer, our understanding of how tumour cells alter the UPS system very early in transformation is rather limited [12]. The identification of essential and druggable key players within this class of enzymes has the potential to hold novel therapeutic strategies. Deubiquitinating enzymes are such a therapeutically promising class of enzymes, as individual members can be targeted by small‐molecule inhibitors [13, 14, 15].

In this study, we report that the deubiquitylase USP28 presents a UPS enzyme, which is commonly upregulated during early stages of oncogenic transformation in lung cancer. Irrespective of oncogenic driver, tumour cells upregulate USP28, which stabilizes proto‐oncogenes, such as c‐MYC, c‐JUN or NOTCH. Tumour cells are addicted to USP28 to allow oncogenic transformation, and its inhibition via the small‐molecule inhibitor AZ1 [16] partially reverts the oncogenic transformation. Finally, combining USP28 targeting with targeted therapy against commonly found oncogenic drivers potentiates treatment responses, at least *in cellulo*, indicating that the UPS system, exemplified by USP28, is a promising target structure for lung cancer.

## 2 Materials and methods

### 2.1 Cell lines

Human basal bronchial epithelial BEAS‐2B cells were originally transformed with SV40 large T antigen [17]. The cell line BEAS‐2B was a kind gift of M. A. Calzado Canales (Universidad de Córdoba, Hospital Reina Sofia, Córdoba, Spain). BEAS‐2B oncogenic cells were generated upon retroviral infection of BEAS‐2B differentiated (DIF) with the next plasmids: EGFR (addgene number: #11011), EGFR L858R (addgene number: #11012), pBabe puro HA PIK3CA (addgene number: #12522), pBabe puro HA PIK3CA H1047R (addgene number: #12524), pBabe puro HA PIK3CA E545K (addgene number: #12525), pBabe puro HRAS G12D (HRAS G12D was cloned into pBabe puro in our laboratory) and pBabe puro BRAF V600E (addgene number: #15269). The plasmids EGFR and EGFR L858R were a gift from M. Meyerson [18]. pBabe puro HA PIK3CA H1047R, HA PIK3CA E545K and HA PIK3CA were a gift from J. Zhao [19]. pBabe Puro BRAF V600E was a gift from W. Hah [20] For virus production, HEK293‐T cells were used. Cell lines used in this publication are listed in Table S1.

### 2.2 Tissue culture reagents and drugs

Cells were plated on Greiner dishes and incubated at 37 °C, 95% relative humidity and 5% CO_2_ in a cell incubator for optimal growth conditions. DIF BEAS‐2B, oncogenic BEAS‐2B and HEK‐293T cells were cultured in DMEM (Gibco; ThermoFischer, Darmstadt, Germany) supplemented with 10% FBS/1% Pen‐Strep. Undifferentiated (UD) cells were cultured in LHC‐9 (Gibco) supplemented with 1% Pen/Strep. To cultivate UD BEAS‐2B cells, the dishes were precoated with precoating solution composed of: 0.03% collagen (in 0.1 m acetic acid), 0.01% fibronectin and 0.001% BSA. UD cells were supplemented with 10% FBS to induce preoncogenic differentiation. The cells were routinely tested for mycoplasma via PCR. The reagents and drugs were dissolved in DMSO. AZ1, gefitinib, buparlisib and vemurafenib were purchased from Selleckchem (Distributed in Germany via Absource Diagnostics, Munich, Germany). Drugs and reagents are listed in Table S1.

### 2.3 AAV, retrovirus and lentivirus production and purification

Adeno‐associated viruses (AAVs) were generated and packaged in HEK293‐T cells seeded in 15‐cm cell culture dishes (60–70% confluence). Cells were transfected with the plasmid of interest (10 μg), pHelper (15 μg) and pAAV‐DJ (10 μg) using PEI in ratio 2 : 1 (70 μg). After 96 h, AAV isolation from cells was performed as previously described [21]. For retrovirus production, HEK293 cells (70% confluence) were transfected with the Babe plasmid of interest (15 μg), pUMVC (10 μg) and VSV‐G (10 μg) using PEI (70 μg). After 96 h, the medium containing retrovirus was filtered (0.45 µm) and stored at −80 °C. For lentivirus production, HEK293 cells (70% confluence) were transfected with the plasmid of interest (15 μg), pPAX (10 μg) and pPMD2 (10 μg) using PEI (70 μg). After 96 h, the medium containing lentivirus was filtered (0.45 µm) and stored at −80 °C.

### 2.4 *In* *vitro* DNA transfection and infection

DNA transfection was performed exposing 60% confluence BEAS‐2B cells plated in a 6‐well cell culture dish to a mix of 2.5 μg plasmid of interest, 200 μL DMEM‐free serum and 5 μL PEI (1 : 2 ratio). Upon 6‐h incubation at 37 °C, 5% CO_2_ and 95% relative humidity, the medium was removed and substituted by DMEM (Gibco) supplemented with 10% FBS/1% Pen‐Strep. For viral infection, 10 MOI (multiplicity of infection) of retroviruses (LVs) was added to normal medium of the cells in the presence of polybrene (5 μg·mL^−1^). The cells exposed to the viruses were incubated at 37 °C, 5% CO_2_ and 95% relative humidity for 4 days. The infected cells were identified and selected by exposure to 2.5 μg·mL^−1^ puromycin for 72 h.

### 2.5 RT‐PCR

RNA was isolated with PeqGOLD Trifast (Peqlab; VWR, Karlsruhe, Germany), as indicated in the manufacturer's instructions. RNA was reverse‐transcribed into cDNA using random hexanucleotide primers and M‐MLV enzyme (Promega, Walldorf, Germany). Quantitative RT‐PCR was performed with SYBR Green Mix (ABgene via ThermoFischer) on the instrument ‘Step One Real‐time Cycler’ (ABgene) The RT‐PCR program employed in this research is as follows: 95 °C for 15 min, 40× (95 °C for 15 s, 60 °C for 20 s and 72 °C for 15 s), 95 °C for 15 s and 60 °C for 60 s. Relative expression was generally calculated with the ΔΔ*C*_t_ relative quantification method. Melt curve was performed for all primers. For visualization purposes, Excel (Microsoft, Redmond, WA, USA) was used for data analysis and Affinity Designer for graphical presentation. Primers used for this publication are listed in Table S1.

### 2.6 Plasmids, sgRNA and shRNA design

Single‐guide RNAs (sgRNAs) were designed using the CRISPR online tool: https://zlab.bio/guide‐design‐resources). shRNAs were designed using SPLASH‐algorithm: http://splashrna.mskcc.org/) or RNAi Consortium/Broad Institute: www.broadinstitute.org/rnai‐consortium/rnai‐consortium‐shrna‐library. Oligonucleotides used in this publication are listed in Table S1.

### 2.7 Operetta analysis, immunofluorescence, cell viability, in cell western blot, Bliss synergy and GI50

The number of cells was quantified using Operetta High‐Content Imaging System (PerkinElmer, Rodgau, Germany) (number of DAPI‐positive cells) or Invitrogen (ThermoFischer) Countess II FL (number of cells after trypsinization) upon indicated treatments. For the Operetta High‐Content Imaging System, cells were seeded in 384‐well plates at equal density and exposed to indicated treatments. Then, the cells were fixed using 4% PFA for 10 min and then permeabilized using 0.5% Triton X‐100 in PBS for 5 min. For immunofluorescence (IF), primary antibodies (1/100) were incubated ON at 4 °C, followed by subsequent incubation with the secondary antibody (1/300) for 1 h at room temperature. After antibody exposure, samples were washed twice with PBS. Before quantification, the cells were stained with DAPI (Thermo Fisher, Darmstadt, Germany). For the quantification of dead cells, 1 µg·mL^−1^ PI was added to the cell medium of live cells for 20 min upon indicated treatments. For quantification of dead cells, 1 µg·mL^−1^ PI (Merck, Taufkirchen, Germany) was added to the cell medium of live cells for 20 min upon indicated treatments. For quantification of proliferative cells, samples were subjected to Ki‐67 (Santa Cruz ab: sc‐23900; 1/100; Santa Cruz Biotechnology, Heidelberg, Germany) staining by IF before imaging. A number of dead, proliferative and total cells were determined counting the number of positive nucleus for PI, Ki‐67 or DAPI with the Harmony software (PerkinElmer).

For *in cell* western blotting, 4000 cells were seeded in 96‐well standard tissue culture adherent plates (Greiner bio‐one, Frickenhausen, Germany) and cultured in their respective medium overnight. Next day, the cells were exposed to various treatment regiments until experimental endpoint. At endpoint, the medium was aspirated and cells were fixed with ice‐cold (−20 °C) methanol on ice for 10 min. Methanol was removed, and the cells were washed three times in PBS (5 min each), followed by blocking in 5% albumin (fraction V; Carl Roth, Karlsruhe, Germany), and dissolved in PBS for 30 min. Blocking solution was removed and primary labelled antibody solution (Lamin A/C 790, Santa Cruz, sc‐376248 AF790, 1/500 dissolved in 5% BSA/PBS) added onto cells, followed by an overnight incubation at 4 °C. Next, the samples were washed three times in PBS (10 min each), and fluorescence staining was imaged using a LiCor CLx Immunoblotter (PerkinElmer). The total fluorescent intensity per well was assessed using the image studio software (PerkinElmer).

Bliss synergy was calculated using the total number of cells upon indicated treatments. For calculation of synergy, the combenefit software was previously described [22]. GI50_50_ was generated using the online tool: www.aatbio.com. For crystal violet cell viability, the cells were stained with 0.5% crystal violet and analysed using the imagej software (staining intensity is between 0 and 255). For visualization purposes, Excel (Microsoft) and Affinity Designer were used as bioinformatic tools. The antibodies used in this publication are listed in Table S1.

### 2.8 Immunoblot

The cells were lysed in RIPA lysis buffer (20 mm Tris/HCl, pH 7.5, 150 mm NaCl, 1 mm Na^2^EDTA, 1 mm EGTA, 1% NP‐40 and 1% sodium deoxycholate), supplemented with proteinase inhibitor (1/100) via sonication with VWR Branson 250 Sonifier (VWR, Darmstadt, Germany) (duty cycle at 20% and output control set on level 2; 10 sonication/1 min cycles per sample). Fifty micrograms protein was boiled in 5× Laemmli buffer (312.5 mm Tris/HCl, pH 6.8, 500 mm DTT, 0.0001% bromophenol blue, 10% SDS and 50% glycerol) for 5 min and separated on 10% Tris gels in the running buffer (1.25 m Tris base, 1.25 m glycine and 1% SDS). After separation, the protein was transferred to polyvinylidene difluoride membranes (Immobilon‐FL) in transfer buffer (25 mm Tris base, 192 mm glycine and 20% methanol). Membrane was exposed to blocking buffer (0.1% casein, 0.2× PBS and 0.1% Tween‐20) for 45–60 min at room temperature (RT). Then, the membranes were incubated with primary antibody (1/1000) in a buffer composed of 0.1% casein, 0.2× PBS and 0.1% Tween‐20 for 6 h at room temperature (RT). The membrane was incubated with indicated secondary antibody (1/10 000) in a buffer composed of 0.1% casein, 0.2× PBS, 0.1% Tween‐20 and 0.01% SDS for 1 h at RT. The membranes were recorded in Odyssey^®^ CLx Imaging System and analysed using the image studio software (LiCor Sciences, Bad Homburg, Germany). The antibodies used in this publication are listed Table S1.

### 2.9 Ubiquitin suicide probe/warhead DUB activity assays

The cells were resuspended in HR buffer (50 mm Tris/HCl, pH 7.4, 5 mm MgCl_2_, 250 mm sucrose and 0.1% NP‐40), supplemented with protease inhibitor. Lysis was performed by three freeze–thaw cycles. Twenty‐five micrograms of cell lysate was transferred to a new Eppendorf tube, and 3 µL of a 1 : 1 : 1 mixture of Ub‐VME, Ub‐VS and Ub‐PA suicide probes (UbiQ) resuspended in 50 mm NaOAc and 5% DMSO was added to the mixture. In order to adjust the pH, 50 mm NaOH was added. Then, the samples were mixed and incubated for 1 h at 37 °C shaking. After addition of Laemmli buffer, the samples were boiled for 5 min and immunoblotting was performed.

### 2.10 Human lung cancer samples

Human samples were obtained from Pathology Department, Córdoba (Spain), Pathology Department University Hospital Würzburg (Germany) and U.S. Biomax (lung microarray slides; slide LC2083). Informed and written consent was obtained from all patients. Experiments were in agreement with the principles set out in the WMA Declaration of Helsinki and the Department of Health and Human Services Belmont Report. Samples are approved under ethics approval licence decret 439/2010 (Hospital Universitario Reina Sofía) and ethics approval 17/01/2006 (University Hospital Würzburg).

### 2.11 Analysis of human publicly available data sets

Oncoprints were generated using the cBioPortal online tool. Briefly, Oncoprints generate graphical representations of genomic alterations, somatic mutations, copy‐number alterations and mRNA expression changes. Graphical representations of somatic mutations were performed using the online tool cBioPortal. TCGA data were used for the different analysis. Correlation analysis and USP28 expression analysis in different subtypes of adenocarcinoma (ADC) and squamous cell carcinoma (SCC) lung tumours were performed using GEPIA's software [23]. For GEPIA gene expression, the differential analysis was based on: ‘TCGA tumours vs (TCGA normal)’, whereas the expression data were log2(TPM + 1)‐transformed and the log2FC was defined as median (tumour) – median (normal). *P*‐values were calculated with a one‐way ANOVA comparing tumour with normal tissue.

The online tool KMplot [24] was used to analyse different types of survival and generate Kaplan–Meier curves based on gene expression data from microarrays obtained from GEO, caBIG and TCGA. Using the KM plotter online tool, patients were split using the option ‘Auto select best cutoff’ in high or low USP28 gene expression groups. *P*‐values for log‐rank tests of the Kaplan–Meier curves were calculated with the online tool KM plotter. Depmap (version 2020) was used to analyse and visualize Pearson's correlation between the genetic expression of USP28 and BRAF/AKT2 in cancer cell lines. *P*‐value and linear regression were calculated by the online tool depmap (https://depmap.org/).

Survival of patients with KRAS‐, EGFR‐, PIK3CA‐ and BRAF‐mutated tumours was analysed using the online tool UCSC Xena [25]. USP28 gene expression of lung cancer samples (including KRAS‐, EGFR‐, PIK3CA‐ and BRAF‐mutated samples) was obtained from TCGA data set. Gene expression was downloaded as log2 (norm_count + 1). Samples were divided in two groups (high and low USP28) based on USP28 expression. The expression of USP28 was defined as high when the respective expression levels were higher than the median expression levels of the analysed data set. The expression of USP28 was defined as low when the respective expression levels were lower than the median expression levels of the analysed data set. Box plots using TCGA and GTEx data were generated using the online tool BoxPlotR [26]. In box plots, the centre line reflects the median and the upper and lower box limits indicate the first and third quartiles. Whiskers extend 1.5× the IQR. For BoxPlotR, the data previously downloaded from UCSC Xena were used to generate the graphics, and *P*‐values were calculated using the two‐tailed *t*‐test. The software packages used for this publication are listed in Table S1.

### 2.12 Animal experiments and histology

All *in vivo* experiments were approved by the Regierung Unterfranken and the ethics committee under the license numbers 2532‐2‐362, 2532‐2‐367, 2532‐2‐374 and 2532‐2‐1003. All animals are housed in standard cages in pathogen‐free facilities on a 12‐h light/dark cycle with *ad libitum* access to food and water. FELASA2014 guidelines were followed for animal maintenance. Veterinarians supervise the welfare of the animals every day. In the presence of pain, stress or suffering, mice were immediately euthanized by cervical dislocation upon isoflurane anaesthesia. The mouse strains used for this publication are listed in the Table S1. The original mouse lines were provided by the JAX repository (Stock No: 028555) and maintained at the animal facility of the Biocenter, University of Würzburg. *In vivo* experiments were carried out in both sexes, and results included in this study are represented as nonsex discriminated.

Adult mice were anaesthetized with isoflurane and intratracheally intubated with 50 μL AAV (3 × 10^7^ PFU) as previously described [21]. Animals were sacrificed by cervical dislocation, and lungs were fixed using 5% NBF. For immunohistochemistry (IHC) and haematoxylin and eosin (H&E), slides were deparaffinized and rehydrated following the previously reported protocol [21]. Briefly, IHC slides were subjected to epitope retrieval and blocked in 3% BSA at RT for 1 h. Antibody manufacturer instructions were followed for every antibody. Yet, in general, primary antibodies (diluted in 1% BSA) were incubated ON at 4 °C followed by three washes with PBS and the subsequent incubation with the DAB secondary antibody for 1 h at RT. Then, the slides were washed twice with 1× PBS for 5 min and stained with the DAB staining solution in 1× PBS. Upon DAB staining, the slides were counteracted with haematoxylin and washed three times with 1× PBS for 5 min. The slides were mounted with 200 μL of Mowiol^®^ 40‐88 covered up by a glass coverslip. IHC slides were recorded using Pannoramic DESK scanner or using the FSX100 microscopy system (Olympus, Hamburg, Germany) and analysed using the case viewer software (3DHISTECH via Sysmex, Norderstedt, Germany), qupath software (University of Edinburgh, Edinburgh, UK) and imagej software. IF samples were recorded using the FSX100 microscopy system (Olympus). The antibodies used in this publication are listed in Table S1.

### 2.13 Organotypic lung tumour slice cultures *ex vivo*

Lung tumours developed upon endotracheal transplantation of KPL cells as previously described [17] or WT lung tissue from WT C57BL6/J‐Rosa26 Sor‐CAGG‐Cas9‐IRES‐eGFP animals were explanted and sectioned in slices using the vibratome. *Ex vivo* slices were relocated in cell culture dishes and maintained in standard cell culture medium (DMEM, 10% FBS) and conditions (37 °C, 5% CO_2_ and 95% relative humidity).

To investigate the genetic modifications induced by CRISPR‐mediated gene targeting, infected slice cultures were propagated for 4 weeks upon infection. Transformed cells attached to the culture plates were further propagated and subjected to Sanger sequencing using site‐specific primers to amplify 1‐kb fragments (around 500 bp proximal and distal to respective sgRNA recognition site).

### 2.14 Sample preparation for mass spectrometry

The sample preparation was performed as described previously. In brief, lysates were precipitated by methanol/chloroform and proteins resuspended in 8 m urea/10 mm EPPS, pH 8.2. The concentration of proteins was determined by the Bradford assay, and 100 µg of protein per samples was used for digestion. For digestion, the samples were diluted to 1 m urea with 10 mm EPPS, pH 8.2, and incubated overnight with 1 : 50 LysC (Wako Chemicals, Düsseldorf, Germany) and 1 : 100 sequencing‐grade trypsin (Promega). Digests were acidified using TFA, and tryptic peptides were purified by tC18 SepPak (50 mg; Agilent, Ratingen, Germany). Ten micrograms peptides per sample were TMT‐labelled, and the mixing was normalized after a single injection measurement by LC‐MS/MS to equimolar ratios for each channel. A bridge channel was prepared by pooling 3 μg from all 24 samples, which were TMT‐labelled together and split into two 10 μg samples for each plex, and 130 µg of pooled peptides was dried for high pH reversed‐phase fractionation.

### 2.15 High pH reversed‐phase fractionation

Labelled peptide samples were pooled, fractionated into eight fractions using the High pH Reversed‐Phase Peptide Fractionation Kit (Thermo Fisher Scientific 84868) according to the manufacturer's protocol and dried. Additionally, for label‐free single shots, 10 µg of peptide is cleaned up with Empore C18 stage tipping and dried right away for shooting.

### 2.16 LC‐MS^3^ proteomics

All mass spectrometry data were acquired in centroid mode on an Orbitrap Fusion Lumos mass spectrometer hyphenated to an easy‐nLC 1200 nano HPLC system using a nanoFlex ion source (Thermo Fisher Scientific) applying a spray voltage of 2.6 kV with the transfer tube heated to 300 °C and a funnel RF of 30%. Internal mass calibration was enabled (lock mass 445.12003 *m/z*). Peptides were separated on a self‐made, 32‐cm‐long, 75 µm ID fused‐silica column, packed in‐house with 1.9 µm C18 particles (ReproSil‐Pur, Dr. Maisch) and heated to 50 °C using an integrated column oven (sonation). HPLC solvents consisted of 0.1% formic acid in water (Buffer A), and 0.1% formic acid and 80% acetonitrile in water (Buffer B).

For total proteome analysis, a synchronous precursor selection (SPS) multinotch MS3 method was used in order to minimize ratio compression as previously described [27]. Individual peptide fractions were eluted by a nonlinear gradient from 3% to 60% B over 150 min followed by a stepwise increase to 95% B in 6 min, which was held for another 9 min. Full‐scan MS spectra (350–1400 *m/z*) were acquired with a resolution of 120 000 at *m/z* 200, maximum injection time of 100 ms and AGC target value of 4 × 10^5^. The most intense precursors with a charge state between 2 and 6 per full scan were selected for fragmentation within 3 s cycle time and isolated with a quadrupole isolation window of 0.7 Th. MS2 scans were performed in the ion trap (Sciex, Darmstadt, Germany) using a maximum injection time of 50 ms and AGC target value of 15 × 10^4^, and fragmented using CID with a normalized collision energy (NCE) of 35%. SPS‐MS3 scans for quantification were performed on the 10 most intense MS2 fragment ions with an isolation window of 1.2 Th (MS) and 2 *m/z* (MS2). Ions were fragmented using HCD with an NCE of 65% and analysed in the Orbitrap with a resolution of 50 000 at *m/z* 200, scan range of 110–500 *m/z*, AGC target value of 1.5 × 10^5^ and a maximum injection time of 150 ms. Repeated sequencing of already acquired precursors was limited by setting a dynamic exclusion of 60 s and 7 p.p.m., and advanced peak determination was deactivated.

### 2.17 Proteomics analysis

Proteomics raw files were processed using proteome discoverer 2.2 (Thermo Fisher). Spectra were recalibrated using the *Homo sapiens* SwissProt database (2020‐03‐12) and TMTpro (+304.207 Da) as static modification at N terminus and lysines, together with carbamidomethyl at cysteine residues. Spectra were searched against human database and common contaminants using Sequest HT with oxidation (M) as dynamic modification together with methionine loss + acetylation and acetylation at the protein terminus. TMTpro (N‐term, K) and carbamidomethyl were set as fixed modifications. Quantifications of spectra were rejected if average S/N values were below 5 across all channels and/or isolation interference exceeded 50%. Protein abundance was calculated by summing all peptide quantifications for each protein. For mixing two plexes, a bridge channel was used additionally. Internal reference scaling (IRS) normalization was performed to obtain proteomics data set across two plexes.

Reactome analysis was performed with PANTHER using the ‘Statistical overrepresentation test’ tool with default settings. For Reactome analysis and violin plots, the common proteins significantly dysregulated in EGFR L858R, BRAF V600E and PIK3CA H1047R BEAS‐2B with respect to DIF. BEAS‐2B cells were selected. Proteins were considered significantly downregulated when *P*‐value < 0.05. *Z*‐score heatmap visualization was performed using morpheus (Broad Institute). The maximum and minimum *Z*‐scores per row used in heatmaps were calculated using Morpheus (Broad Institute). Volcano plots were generating using the software instant clue [28]. Venn diagrams were performed using the online tool: http://bioinformatics.psb.ugent.be/webtools/Venn/. PCA was performed using the online tool CLUSTVIS [29]. Violin plots were generated using the online tool BoxPlotR [26]. For visualization purposes, Excel (Microsoft) and Affinity Designer were used as bioinformatic tools.

### 2.18 Data and software availability

Data are available via ProteomeXchange (PRIDE) with identifier PXD032810.

### 2.19 Contact for reagent and resource sharing

Further information and requests for resources and reagents should be directed to and will be fulfilled by the Lead Contact, Markus E. Diefenbacher (markus.diefenbacher@uni-wuerzburg.de).

## 3 Results

### 3.1 USP28 is expressed in human ‘cell of origin’ for NSCLC and upregulated irrespective of lung tumour subtype

Previous work has identified the ‘cell of origin’ for NSCLC, in particular ADC [BADJ (SPC^+^/CC10^+^) and AT2 (SPC^+^) pulmonary cells] [30, 31], while SCCs, one of the most mutated tumour entities, demonstrated a rather high degree of plasticity and flexibility regarding the required cell of origin [32]. Here, the genetic driver combination was the determining factor [33].

**Fig. 1:**
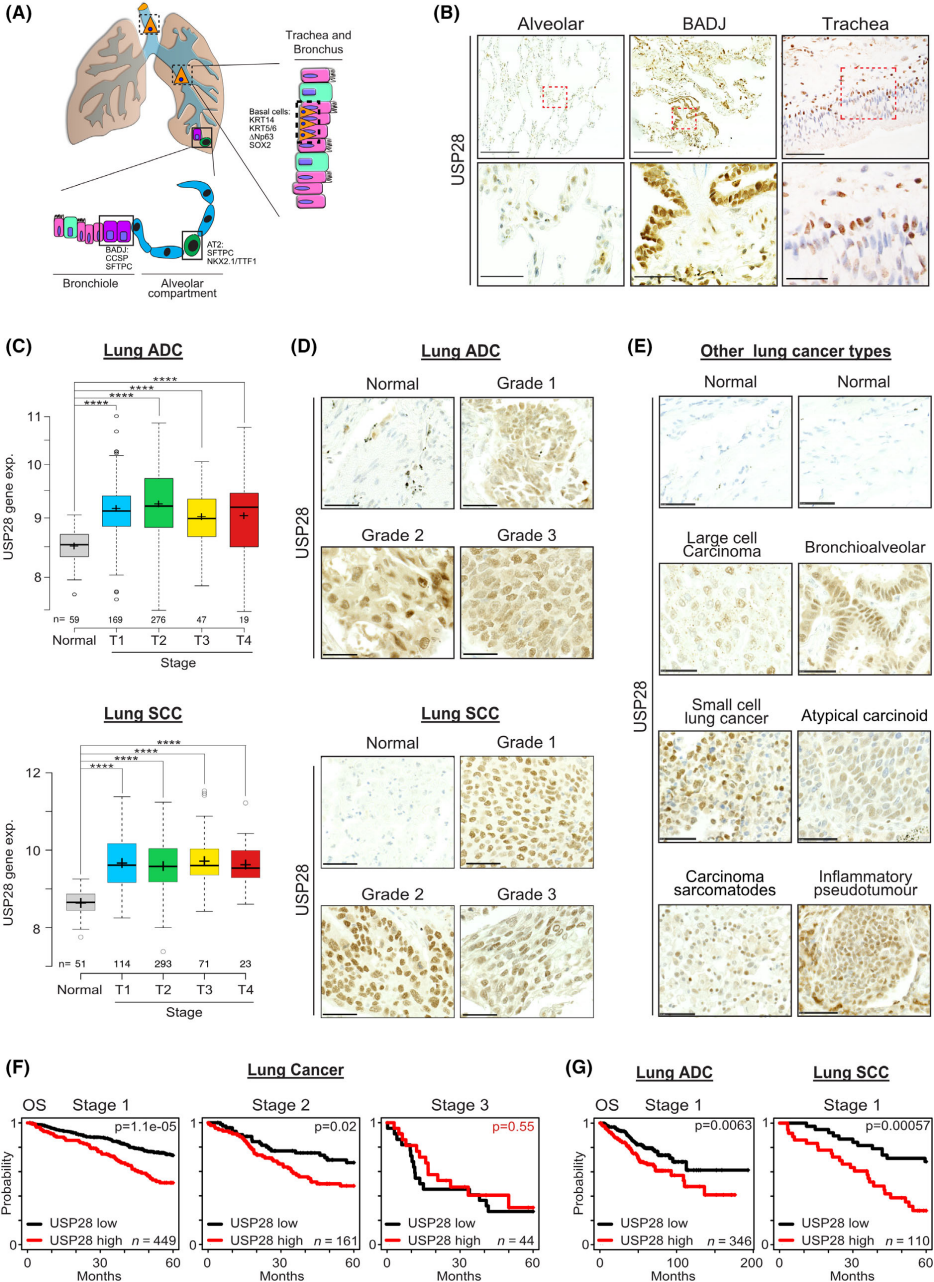
USP28 is expressed in human ‘cell of origin’ for NSCLC and upregulated irrespective of lung tumour subtype. (A) Schematic representation of the cellular composition of the tracheal, bronchoalveolar and alveolar compartment. Highlighted are the respective tissue‐residing stem cells. Trachea = basal cells, Bronchoalveolar duct junction = BADJ (bronchoalveolar duct junction) stem cells, alveolar compartment = AT2 (alveolar type II cells) cells. (B) Immunohistochemical staining of endogenous USP28 in patient lung‐resected material. Shown are representative images of alveolar, bronchial and tracheal sections. Red boxes were magnified in the lower panel. Upper scale bar = 200 µm. Lower scale bar = 50 µm. *n* = 200. (C) Expression of USP28 in nontransformed and NSCLC, ADC and SCC samples, relative to tumour stage (T1–T4). Publicly available data from TCGA data set obtained from http://xena.ucsc.edu/. *P*‐values were calculated using the two‐tailed *t*‐test statistical analysis. *P < 0.05, **P < 0.01, ***P < 0.001 and ****P < 0.0001. In box plots, the centre line reflects the median and the upper and lower box limits indicate the first and third quartiles. Whiskers extend 1.5× the IQR. Plot was generated using the online tool http://shiny.chemgrid.org/boxplotr/. (D) Immunohistochemical staining of USP28 on human NSCLC tissue microarrays from www.biomax.us (slide LC2083: lung disease spectrum). Samples were ranging from grades 1 to 3, and histological classification (ADC or SCC) was defined. Where applicable, nontransformed tissue was included. Shown are representative images per tumour type. Tissue microarray contains 208 samples (to obtain more information about the samples, please visit www.biomax.us slide number LC2083). Scale bar = 50 µm. *n* = 208. (E) Immunohistochemical staining of USP28 on human lung cancer tissue microarrays of various lung cancer subtypes arrays from www.biomax.us (slide LC2083: lung disease spectrum). Where applicable, nontransformed tissue was included. Shown are representative images per tumour type. Tissue microarray contains 208 samples (to obtain more information about the samples, please visit www.biomax.us slide number LC2083). Scale bar = 40 µm. *n* = 208. (F) Kaplan–Meier plots of NSCLC patient overall survival (OS), relative to USP28 expression, at Stage 1 (*P* = < 0.0005), Stage 2 (*P* = 0.02) to Stage 3 (*P* = 0.55). Data were generated using the online tool www.kmplot.com. *P*‐values were calculated using survival log‐rank statistical test. (G) Kaplan–Meier plots of NSCLC ADC and SCC patient OS, relative to USP28 expression, at Stage 1 (ADC *P* = 0.0063; SCC *P* = 0.00057). Data were generated using the online tool www.kmplot.com. *P*‐values were calculated using survival log‐rank statistical test. See also Fig. S1.

We previously observed that genetic loss of USP28 affected tumour burden in a murine *in vivo* NSCLC model [21]. To investigate whether this could be attributed to the expression of USP28 in the stem cell/cell‐of‐origin compartment of NSCLC, we performed IHC against USP28 in human lung tissue samples. High USP28 expression was detectable in the tracheal basal cells (triangular‐like shape), BADJ and AT2 cells, the ‘cells of origin’ for NSCLC (Fig. 1A,B). Next, we were intrigued if USP28 expression is altered relative to tumour type and/or grade. To address this question, we analysed publicly available gene expression data sets of NSCLC patients (Fig. 1C). *USP28* was found to be upregulated already at an early stage in lung cancer, when compared to wild‐type tissue (Fig. 1C). Furthermore, *USP28* upregulation is a common feature of tumour cells, irrespective of histological or molecular tumour subtype (Fig. 1C, Fig. S1A,B). This was further validated by immunostaining of nontransformed versus tumour samples from NSCLC patients, where a significant increase in USP28 protein abundance was detected already at low‐grade stages for lung ADC and SCC (Fig. 1D,E).

Analysing survival data and correlating USP28 expression to tumour stages, it became clear that, especially at early stages, USP28^high^ expressing tumours significantly correlated with an overall shortened survival (Fig. 1F), and this observation was independent of tumour subtype (Fig. 1G).

These data indicated that USP28 is upregulated upon oncogenic transformation and that upregulation appears to be an early event in the tumorigenesis of lung and holds prognostic value.

### 3.2 USP28 is expressed in murine lung stem cells and required to establish oncogenic transformation *in vivo*

To investigate that USP28 is required for NSCLC induction and its upregulation occurs at early stages, we utilized CRISPR/Cas9 genetic engineering mouse models of NSCLC [21, 34].

**Fig. 2:**
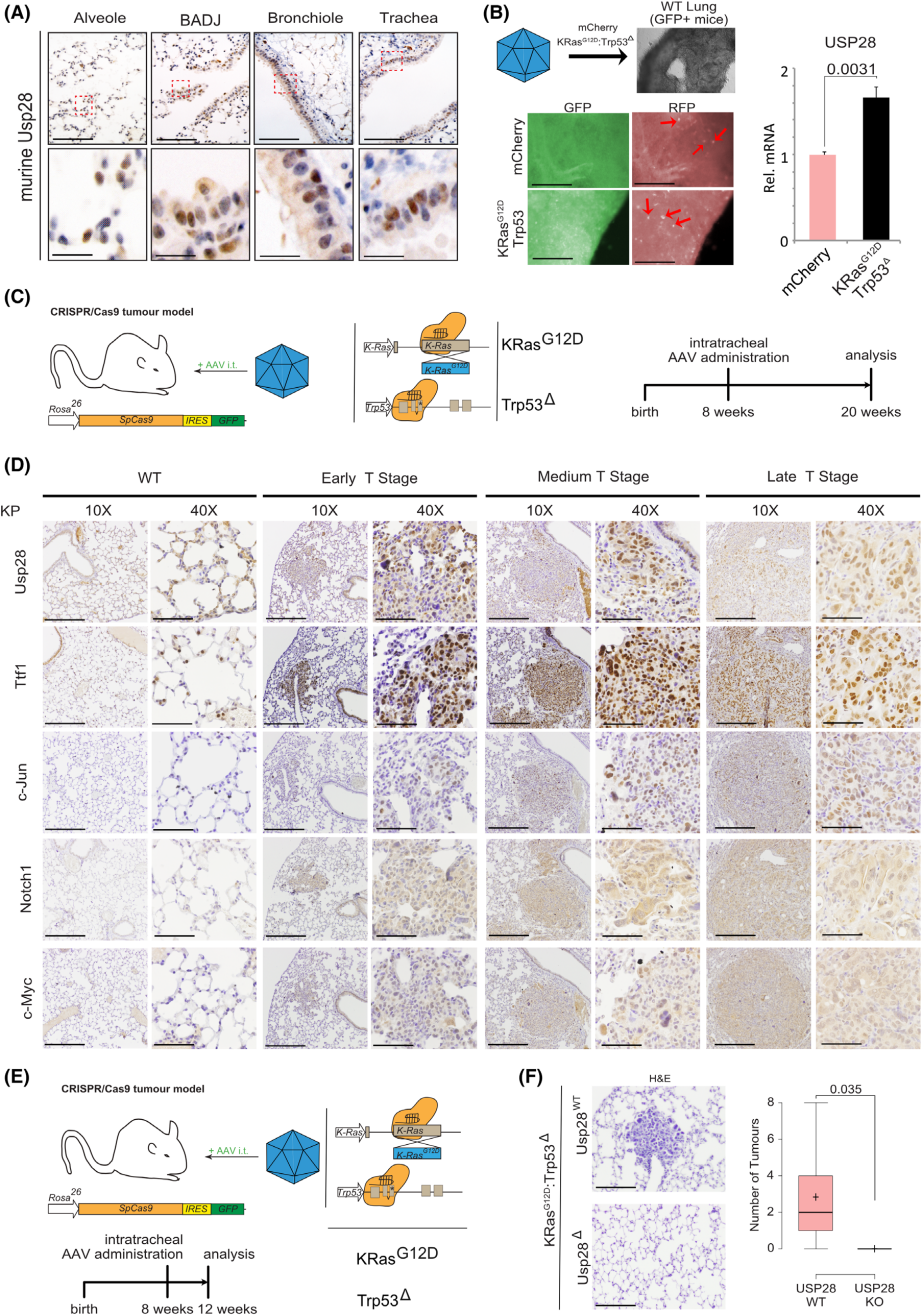
USP28 is expressed in murine lung stem cells and required to establish oncogenic transformation *in vivo*. (A) Immunohistochemical staining of endogenous Usp28 in murine lung wild‐type tissue. Shown are representative images of alveolar, bronchial, bronchoalveolar duct junction and tracheal sections. Red boxes indicate highlighted areas. Upper scale bar = 100 µm. Lower scale bar = 15 µm. *n* = 3. (B) *Ex vivo* onset of oncogenic transduction by CRISPR‐mediated gene editing and deletion of *Trp53* and mutation of *KRas* to *KRas^G12D^* (KP) upon AAV infection of organotypic lung slice cultures. Lung slice cultures were generated from *C57BL6/J‐Rosa26^Sor‐CAGG‐Cas9‐IRES‐eGFP^* mice, and AAV encodes mCherry as marker. Fluorescent images of lung slice cultures postinfection with AAV. GFP = lung; RFP = AAV‐infected lung epithelial cells. Red arrows indicate mCherry‐positive infected cells. Tissue sections were harvested and subjected to RNA isolation, followed by RT‐PCR analysis of *Usp28* mRNA expression in control and KP‐infected slices. *n* = 3. *P*‐values were calculated using the two‐tailed *t*‐test statistical analysis. Scale bar = 350 µm. Quantitative graphs are represented as mean ± SD (standard deviation). (C) Schematic representation of *in vivo* CRISPR gene editing to delete *Trp53* and mutate *KRas* to *KRas^G12D^* (KP) upon intratracheal administering of AAV. Animals are sacrificed 12 weeks postinfection. (D) Representative images of immunohistochemical staining against endogenous Usp28, Nkx2‐1/Ttf‐1, c‐Jun, Notch1 and c‐Myc in murine lungs infected with either control virus (WT) or KP. Shown are healthy tissues and representative tumours spanning wild‐type early, medium and high T stages, with a low (10×) and high magnification (40×) of individual tumour areas. *n* = 3. Low magnification (10×) scale bar = 200 µm. High magnification (40×) scale bar = 50 µm. (E) Schematic representation of *in vivo* CRISPR gene editing to delete *Trp53*, mutate *KRas* to *KRas^G12D^* (KP) or codelete *Usp28* (KPU) upon intratracheal administering of AAV. Animals are sacrificed 4 weeks postinfection. *n* = 6. (F) Representative H&E staining images for KP and KPU animals 4 weeks postinfection. Quantification of tumour burden per animal. *n* = 6. Scale bar = 100 µm. In box plots, the centre line reflects the median and the upper and lower box limits indicate the first and third quartiles. Whiskers extend 1.5× the IQR. *P*‐values were calculated using two‐tailed *t*‐test statistical analysis. Plot was generated using the online tool http://shiny.chemgrid.org/boxplotr/. See also Fig. S2.

To this end, we analysed the expression pattern of Usp28 by IHC in wild‐type, nontransformed lungs (Fig. 2A). We observed that USP28 showed a comparable expression to human samples (Figs 1B and 2A). Overall, Usp28 expression was elevated in putative stem cells, when compared to surrounding/neighbouring differentiated cells (Fig. 2A).

Next, we wondered whether the transcriptional upregulation of USP28 already occurs at point of transformation. To address this question, we used an *ex vivo* organotypic lung slice culture model. Here, a nontransformed lung from a C53BL6/J‐*Rosa26^Sor‐CAGG‐Cas9‐IRES‐eGFP^* mouse [35] was sectioned into 100‐µm‐thick sections using a vibratome and cultured in standard medium. Twenty‐four hours post sectioning, the slices were infected using an AAV expressing the fluorescent protein mCherry as infection marker, together with sgRNA to delete *Trp53* and mutate endogenous *Kras* to *Kras^G12D^* (KP, Fig. 2B) [21, 34]. As a control vector, we used an mCherry expressing AAV. 7 days post infection, the lung slices were harvested, and mRNA expression of Usp28 was analysed using quantitative PCR (Fig. 2B). Here, *Usp28* expression was significant increased at an early transformative state, when compared to control virus‐infected tissue samples.

Next, we analysed the expression of Usp28 and its substrates c‐Myc, c‐Jun and Notch1 at various grades in murine primary tumours, generated by intratracheal infection with a KP encoding AAV (Fig. 2C). Mice were infected at around 8 weeks of age and sacrificed 12 weeks postinfection. Tumour grade and type were assessed using histopathological means (H&E, Ttf‐1). Already in low‐grade primary tumours, Usp28 and its substrates were significantly upregulated when compared to adjacent, nontransformed lung epithelial tissue (Fig. 2D). The increase in Usp28 and the oncogenic transcription factors persisted in higher grade tumours, as seen by IHC (Fig. 2D), thereby confirming the observation made in patients (Fig. 1C–E, Fig. S1A,B).

Next, we wondered whether the upregulation of Usp28 at an early stage is independent of the oncogenic driver (Fig. S2A,B). For that purpose, we generated murine primary NSCLC using BrafV600E as oncogenic driver. BRAF is genetically altered or transcriptionally changed in 28% of SCC and 25% of ADC lung tumour samples (Fig. S2A). To analyse early‐stage tumours, mice were sacrificed 4 weeks postinfection (Fig. S2B). Tumour grade and type were determined using H&E and immunohistologic stainings against Ttf‐1 and Pcna. Already in early‐stage lung primary tumours, Usp28 was overexpressed when compared to nontransformed tissue (Fig. S2C).

Previously, we reported that loss of Usp28 affected the induction of lung squamous cancer, and its genetic loss affected overall tumour burden [21]. To investigate whether loss of Usp28 affects tumour induction at an early stage or just reduces proliferation of transformed cells, next, we infected constitutive Cas9 expressing mice with an AAV containing either an sgRNA to delete *Trp53* and mutate endogenous *Kras* to *Kras^G12D^* (KP, Fig. 2E) or a virus harbouring additional two sgRNA targeting endogenous Usp28 (KPU, Fig. 2E). Mice were sacrificed 4 weeks postinfection, and while in KP‐infected animals, tumour lesions were detectable, in KPU however, no lesions could be observed (Fig. 2F).

These data suggest that USP28 is expressed in tumour‐initiating cells and is required during early stages of lung cancer transformation independent of tumour subtype or oncogenic driver.

### 3.3 USP28 is increased during early transformation in human BEAS‐2B differentiation assay

**Fig. 3:**
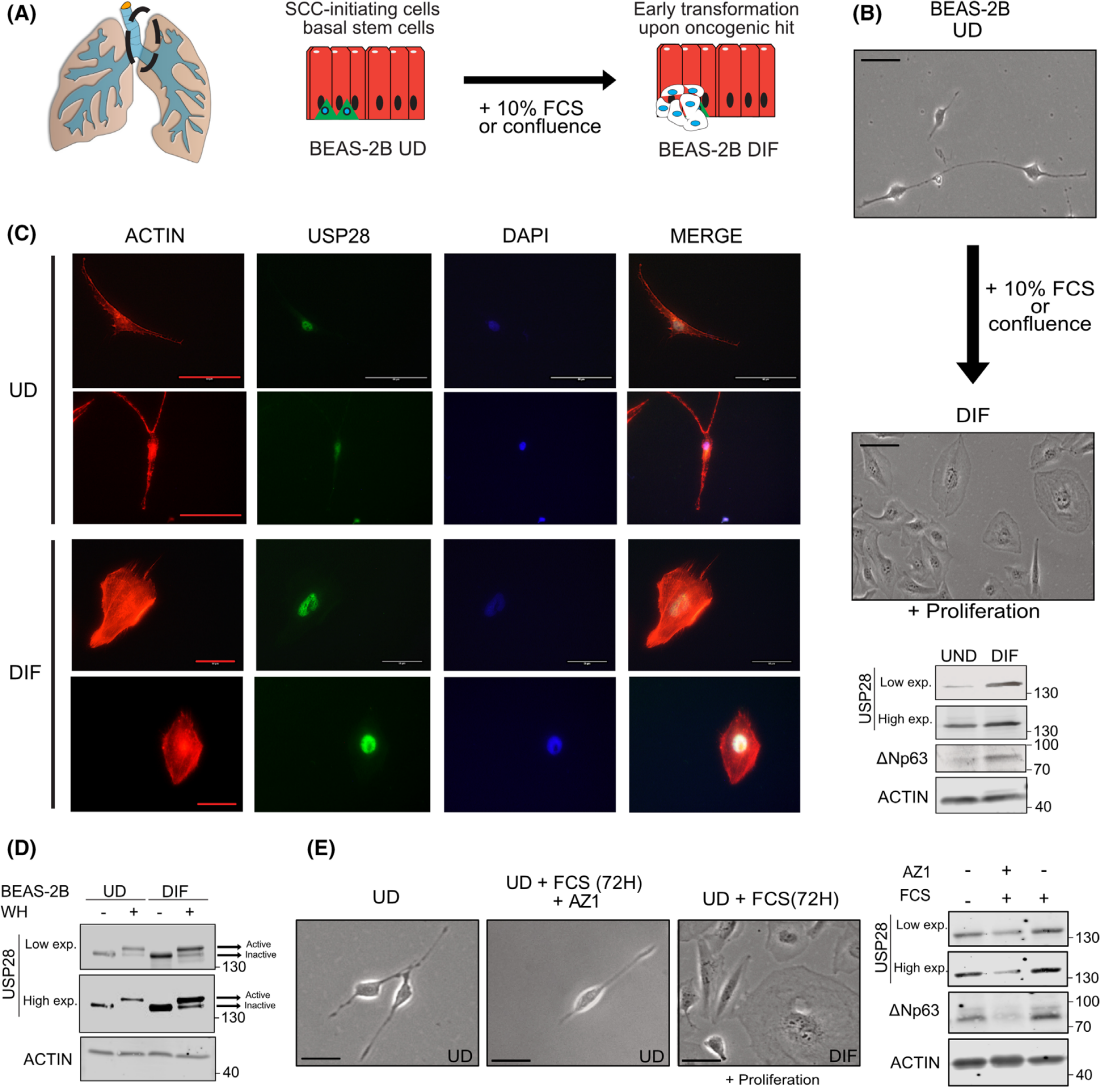
USP28 is increased during early transformation in human BEAS‐2B differentiation assay. (A) Schematic model of the transdifferentiation by culturing stem cell‐like, undifferentiated BEAS‐2B (BEAS‐2B^UD^) in the presence of 10% FBS to induce squamous‐like differentiation, BEAS‐2B^DIF^. (B) Representative bright‐field images of BEAS‐2B before and after serum‐induced transdifferentiation (72 h in the presence of 10% FBS, UD to DIF). Immunoblot of *n* = 10 against endogenous USP28 and ∆NP63 of BEAS‐2B^UD^ and BEAS‐2B^DIF^. ACTIN served as loading control. Representative blot of *n* = 3. Scale bar = 30 µm. (C) Immunofluorescence of endogenous USP28 prior to and postdifferentiation. USP28 in green, ACTIN in red and DAPI as nuclear counterstain. *n* = 50 cells. Scale bar = 50 µm. (D) Immunoblot against endogenous USP28 in the absence or presence of an ubiquitin suicide probe (warhead, WH) to assess USP28 enzymatic activity in undifferentiated and differentiated BEAS‐2B cells. Upon binding to the activity probe, a shift in molecular weight is observed, indicative of enzymatic activity (see arrows). ACTIN served as loading control. Representative immunoblot of *n* = 3. (E) Representative bright‐field images of BEAS‐2B prior to and postculture in serum‐induced transdifferentiation conditions, in the presence or absence of the USP28 inhibitor AZ1 (15 µm, 72 h). *n* = 50 cells. Immunoblot against endogenous USP28 and ∆NP63 of BEAS‐2B^UD^ exposed to either 10% FBS or 10% FBS and 15 µm AZ1. Scale bar = 15 µm. Actin served as loading control. Representative blot of *n* = 3. Exp., exposure; WH, ubiquitin suicide warhead probe. See also Fig. S3.

As it appears that transcriptional upregulation and increased protein abundance of USP28 are required early events in oncogenic transformation, we used a human cell line system to recapitulate these early events. The immortalized human tracheal cell line BEAS‐2B retains the ability to grow as a progenitor‐like cell, but in a cell density‐dependent fashion or under the exposure of FBS can terminally differentiate into a squamous‐like, preoncogenic and highly proliferative cell (Fig. 3A) [36, 37]. Indeed, culturing these cells under progenitor specific culturing conditions maintained the cells in a ‘spindle‐like’ shape as previously reported (Fig. 3B and Fig. S3A; undifferentiated BEAS‐2B; BEAS‐2B^UD^). Upon FBS exposure, BEAS‐2B^UD^ cells start to alter their morphology and resemble a squamous phenotype (Fig. 3B,C; differentiated BEAS‐2B; BEAS‐2B^DIF^) [36]. Furthermore, USP28 protein abundance was increased in BEAS‐2B^DIF^ when compared to BEAS‐2B^UD^ by IF and immunoblotting against endogenous USP28 (Fig. 3B,C and Fig. S3B). Not only is USP28 upregulated, but the amount of enzymatically active USP28 was increased as well, indicated by the amount of USP28 bound to a ubiquitin suicide probe/warhead (Fig. 3D). Upon preoncogenic differentiation, the known USP28 target ∆Np63 increased in protein abundance, as shown by immunoblotting comparing BEAS‐2B^DIF^ with BEAS‐2B^UD^ (Fig. 3B). To investigate whether the observed differentiation and the consecutive increase in oncoprotein abundance are USP28‐dependent, we exposed BEAS‐2B^UD^ to FBS (Fig. 3E). Upon serum pulse, cells start to differentiate, visualized by bright‐field microscopy, and increase the expression of USP28 and its target ∆Np63 (Fig. 3E). In order to block USP28, we made use of the published USP28 inhibitor AZ1 [16, 21]. The addition of AZ1 resulted in the degradation of USP28 and reduction in ∆Np63, and the cells maintained an undifferentiated morphology (Fig. 3E and Fig. S3C).

These data demonstrate that USP28 is upregulated during premalignant transformation and this leads to an increase in USP28 target oncoproteins.

### 3.4 Oncogenic transformation of BEAS‐2B^DIF^ via EGFR‐PI3K‐MAPK pathway upregulates USP28 and accelerates tumour cell growth

**Fig. 4:**
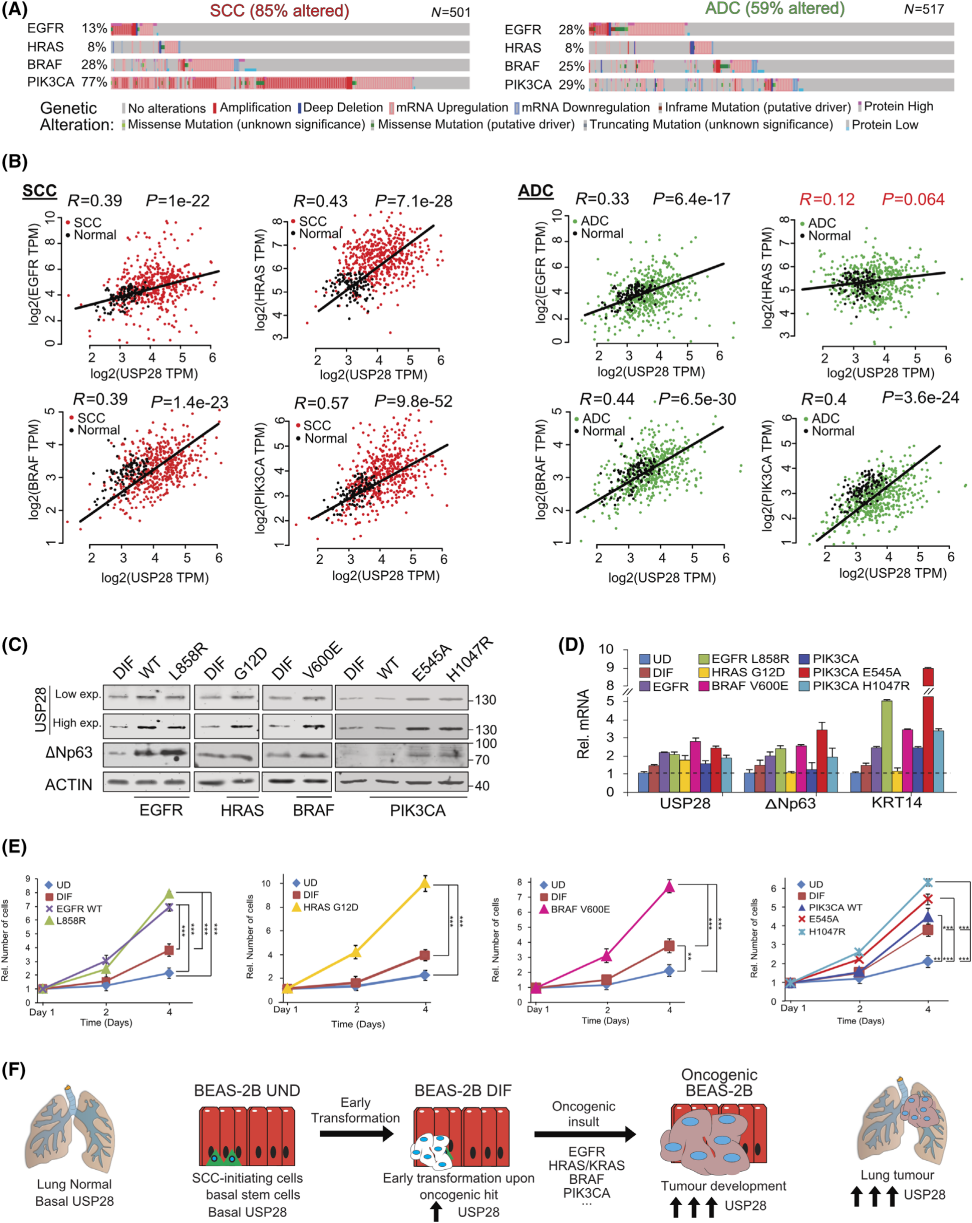
Oncogenic transformation of BEAS‐2B^DIF^ via EGFR‐PI3K‐MAPK pathway upregulates USP28 and accelerates tumour cell growth. (A) Frequently occurring genetic alterations and expression changes in recurring oncogenic drivers found in NSCLC (ADC and SCC). Oncoprints generated with the online tool www.cbioportal.org. (B) mRNA expression of Spearman's correlation between USP28 and EGFR, HRAS, BRAF or PIK3CA in NSCLC (SCC and ADC). Correlation and *P*‐value generated with the online tool GEPIA www.gepia2.cancer‐pku.cn/ using lung cancer TCGA publicly available data set. (C) Immunoblot against endogenous USP28 and the oncogenic transcription factor ∆NP63 in either BEAS‐2B^DIF^ or BEAS‐2B^DIF^ upon retroviral transduction to express the indicated oncogenes EGFR [wild‐type (WT) and L858R], HRAS (G12D), BRAF (V600E) and PIK3CA [wild‐type (WT), E545K and H1047R], respectively. Actin served as loading control. *n* = 3. (D) RT‐PCR of USP28, the SCC transcription factor ∆NP63 and its target cytokeratin 14 (KRT14) in BEAS‐2B^UD^, BEAS‐2B^DIF^ or the various BEAS‐2B^ONC^ as presented in C. Shown are mean log2 fold change expression data, relative to actin, and normalized to the respective expression in BEAS‐2B^UD^ and standard deviation (SD). Shown are mean values and SD of *n* = 3. (E) Relative cell numbers and assessment of growth capacity of BEAS‐2B^UD^, BEAS‐2B^DIF^ or the various BEAS‐2B^ONC^ over a total of 4 days. Cell numbers were analysed at days 1, 2 and 4. Shown are relative mean values and standard deviation. *n* = 3 experiments. *P*‐values were calculated using the two‐tailed *t*‐test statistical analysis. *P < 0.05, **P < 0.01, ***P < 0.001 and ****P < 0.0001. (F) Schematic model of the various stages of oncogenic transformation, as recapitulated by the transdifferentiation from BEAS‐2B^UD^ to BEAS‐2B^DIF^, and from BEAS‐2B^DIF^ to BEAS‐2B^ONC^ (oncogenic transformed BEAS‐2B^DIF^). The observed increases recapitulate the increase in USP28 protein abundance as seen in human lung cancer samples. Exp., exposure; ONC, oncogenic. See also Figs S4 and S5.

During the oncogenic transformation process, cells acquire additional alterations, which are the prerequisite to establish a tumour [38]. Using public data sets, we identified that USP28 expression strongly correlates to the expression of common driver mutations found in NSCLC, encompassing either amplification or mutation of BRAF, EGFR, PI3K and RAS (Fig. 4A,B, Fig. S4A,B). HRAS was the exemption in RAS family, as no correlation with USP28 was observed in ADC; however, NRAS and KRAS positively correlated with USP28 in ADC human samples (Fig. 4B, Fig. S4A,B). To test this observation, we *in vitro* retrovirally transformed BEAS‐2B^DIF^ with various oncogenes (BEAS‐2B^ONC^; Fig. S3C) and investigated the effects on USP28 abundance by immunoblotting and RT‐PCR. As a control, we used a virus only encoding puromycin resistance (Fig. S4D,E). EGFR WT, EGFRL858R, HRASG12D, BRAFV600E, PIK3CA WT, PIK3CAE545A and PIK3CAH1047R expressions were confirmed by immunoblotting and RT‐PCR (Fig. S4D,E). While USP28 was detectable in control‐transformed cells, the expression of oncogenes further increased USP28 levels and mRNA (Fig. 4C,D). Not only was the DUB increased but also the USP28 target protein ∆Np63, along with KRT14, a known ∆Np63 transcriptional target gene (Fig. 4C,D) [39]. It is noteworthy that the overexpression of wild‐type PIK3CA/p110 had little to no effect on overall protein increase (Fig. 4C,D). Here, only the mutant variants E545K and H1047R led to an increase in USP28 and ∆Np63 (Fig. 4C,D). More studies are required to elucidate the differences between functional mutations and amplification of PIK3CA.

Previous studies reported that USP28 is a direct target of c‐JUN and c‐MYC (Fig. S5A) [40, 41]. Since both proto‐oncogenes exert an important role during oncogenic transformation and are increased upon EGFR‐PI3K‐MAPK‐mediated oncogenic transformation [42], we wondered whether the regulation of USP28 in lung cancer depends on these transcription factors as well. Transient transfection of c‐MYC and c‐JUN increased USP28 protein abundance compared with control plasmid transfection in BEAS‐2B cells (Fig. S5B). Furthermore, BEAS‐2B^DIF^ cells showed higher protein abundance for USP28 and its known substrates c‐MYC, c‐JUN and NOTCH1 than BEAS‐2B^UD^ (Fig. S5C) and oncogenic transformation of BEAS‐2B^DIF^ further increased USP28, c‐MYC, c‐JUN and NOTCH1 protein abundance (Fig. S5C).

In summary, c‐MYC, c‐JUN and NOTCH1 are downstream targets of the PI3K‐MAPK pathways and establish a feed‐forward loop in oncogenic transformed cells, contributing to their increased abundance (Fig. S5D). Analysing public data sets revealed that gene expression of the PI3K‐MAPK downstream effectors, AKT2 and BRAF, positively correlated with USP28 in lung cancer cell lines and in various tumour entities (Fig. S5E). Notably, melanoma and tumours arising in liver, eye and bone showed weak correlation between USP28 and AKT2‐BRAF, and for thyroid cancer, we observed a negative correlation. This tumour type presents a tumour entity with overall better prognosis and survival [43].

Next, we wondered whether oncogenic transformation of BEAS‐2B^DIF^ alters cellular growth responses. To investigate potential effects, we compared growth rates of BEAS‐2B^UD^, BEAS‐2B^DIF^ and BEAS‐2B^ONC^ for 4 days (Fig. 4E). While the differentiation already enhanced proliferation and is a consequence of enriched proto‐oncogene abundance (Fig. S4C), upon overexpression of oncogenes, irrespective of oncogenic driver, all generated cell lines demonstrated a significant increase in proliferation, except overexpression of wild‐type PIK3CA/p110 (Fig. 4E).

This observation indicates that USP28 is a downstream target of the PI3K‐MAPK pathway (Fig. 4F) and is contributing to cellular transformation by stabilizing proto‐oncogenes (Fig. S4D).

### 3.5 Malignant transformation renders tumour cells dependent on USP28

**Fig. 5:**
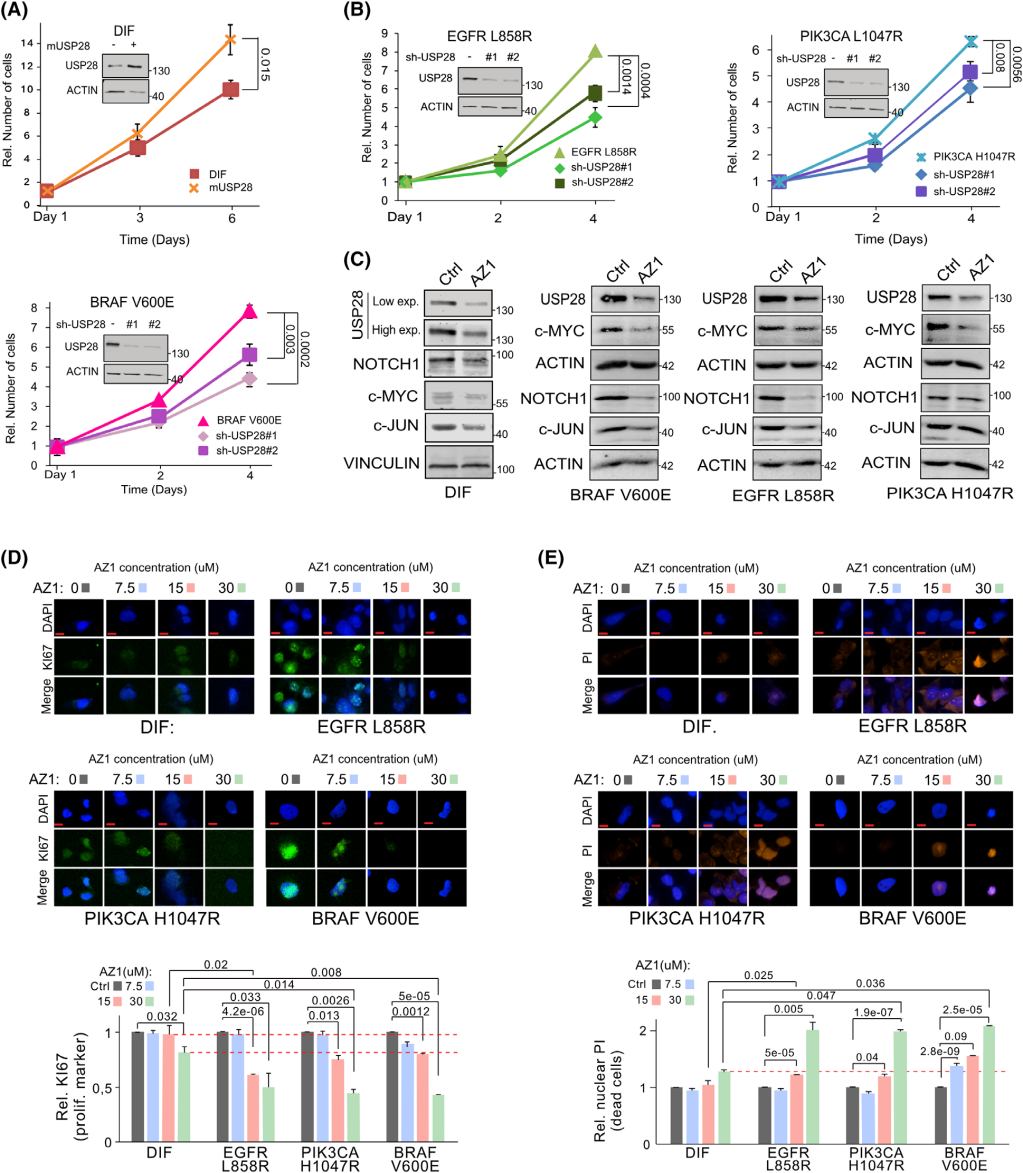
Malignant transformation renders tumour cells dependent on USP28. (A) For growth analysis, BEAS‐2B^DIF^ and mUSP28 (BEAS‐2B^DIF^ transduced with a conditional murine USP28 overexpression plasmid) cells were precultured for 72 h in the presence of 1 µg·mL^−1^ doxycycline, followed by reseeding and counting of cells at Day 1, Day 3 and Day 6. Shown are mean values and standard deviation (SD) of *n* = 3. *P*‐values were calculated using the two‐tailed *t*‐test statistical analysis. For control western blot, BEAS‐2B^DIF^ control and mUsp28 cells were cultured in the presence of 1 µg·mL^−1^ doxycycline for 72 h prior to immunoblotting. Immunoblot showing protein abundance of USP28 in BEAS‐2B^DIF^ upon lentiviral transduction with either a control or a doxycycline‐inducible overexpression of murine Usp28. Actin served as loading control. *n* = 3. (B) For growth analysis, cells were seeded at equal cell density and counted at Day 1, Day 2 and Day 4. Shown are mean values and standard deviation of *n* = 3. *P*‐values were calculated using the two‐tailed *t*‐test statistical analysis. Immunoblot showing protein abundance of USP28 in oncogenic transduced BEAS‐2B^ONC^ (EGFRL858R, PIK3CA L1047R and BRAFV600E) upon lentiviral transduction with either a control or two individual constitutive shRNA targeting USP28. Actin served as loading control. *n* = 3. (C) Immunoblots against endogenous USP28 and its substrates NOTCH1, c‐MYC and c‐JUN in either BEAS‐2B^DIF^ or oncogenic transduced BEAS‐2B^ONC^ (BRAFV600E, EGFRL858R and PIK3CA L1047R) upon exposure to either DMSO or 15 µm AZ1 for 24 h. Vinculin and actin served as loading control. *n* = 3. (D) Immunofluorescence of Ki‐67 expression in BEAS‐2B^DIF^ or oncogenic transduced BEAS‐2B^ONC^ (EGFRL858R, PIK3CA L1047R and BRAFV600E) cultured in the presence of increasing concentrations of AZ1 [0 (DMSO), 7.5, 15, and 30 µm] for 72 h to assess effects in cell proliferation. Shown are representative cells. Quantification of Ki‐67 expression in 30–45 20× fields from three independent wells per condition. *n* = 3 DAPI served as nuclear marker. Red dashed lines reflect the difference between BEAS‐2B^DIF^ and BEAS‐2B^Onc^ (EGFRL858R, PIK3CA L1047R and BRAFV600E) upon exposure to 15 and 30 µm AZ1. Shown are mean values and standard deviation (SD). *P*‐values were calculated using the two‐tailed *t*‐test statistical analysis. Scale bar = 5 µm. (E) Immunofluorescence of propidium iodide (PI) incorporation in BEAS‐2B^DIF^ or oncogenic transduced BEAS‐2B^ONC^ (EGFRL858R, PIK3CA L1047R and BRAFV600E) cultured in the presence of increasing concentrations of AZ1 [0 (DMSO), 7.5, 15, and 30 µm] for 72 h to assess effects on cell death and apoptosis. Shown are representative cells. Quantification of PI‐positive cells in 30–45 20× fields from three independent wells per condition. *n* = 3. DAPI served as nuclear marker. Shown are mean values and standard deviation. Red dashed line reflects the difference between BEAS‐2B^DIF^ and BEAS‐2B^Onc^ (EGFRL858R, PIK3CA L1047R and BRAFV600E) upon exposure to 30 µm AZ1. *P*‐values were calculated using the two‐tailed *t*‐test statistical analysis. Scale bar = 5 µm. Ctrl, control; Exp, exposure; ONC, oncogenic. See also Fig. S6.

As USP28 was upregulated in an oncogene‐dependent fashion, we wondered whether the increase in USP28 contributes to the proproliferative phenotype. The inducible overexpression of murine USP28 was sufficient to increase the proliferation of BEAS‐2B^DIF^ to a comparable extent than PIK3CA mutant cells (Fig. 5A). Conversely, using two independent shRNA sequences to target USP28 expression, irrespective of oncogenic driver, loss of USP28 impaired the proliferation of transformed cells (Fig. 5B). This was further confirmed using the KRAS G12S mutant lung cancer cell line A549 (Fig. S6A). Depletion of USP28 by two independent shRNA sequences strongly reduced the overall abundance of MYC and the proliferation marker PCNA (Fig. S6B). As a consequence, A549 proliferation was significantly reduced (Fig. S6C). Next, we wondered whether the small‐molecule inhibitor AZ1, which impairs USP25/28 enzymatic activity, affects proliferation of oncogenic transformed BEAS‐2B. Furthermore, we wanted to address whether the inhibitor shows selectivity towards specific oncogenic drivers. To this end, BEAS‐2B^DIF^ and BEAS‐2B^ONC^ cells were grown in the presence of increasing concentration of AZ1, followed by calculating the half‐maximal inhibitory concentration (IC_50_) by assessing cell numbers. It was revealed that EGFRL858R‐ and BRAFV600E‐transduced BEAS‐2B^ONC^ cells tolerated ~ 16 µm AZ1, while cells transformed by PIK3CAH1047R required ~ 20 µm (Fig. S6D). Given the rather comparable IC_50_ concentrations, we treated BEAS‐2B^DIF^ and BEAS‐2B^ONC^ cells with 15 µm AZ1 for 24 h, followed by immunoblotting against USP28 and its substrates NOTCH1, c‐MYC and c‐JUN (Fig. 5C). Upon exposure to AZ1, all cell lines showed a marked reduction in the protein abundance of USP28 and its target substrates (Figs 3E and 5C). Next, we investigated whether AZ1 impairs cell proliferation or increases cell death in a dose‐dependent fashion, and whether AZ1 affects all cells or is limited to oncogenic transformed cells (Fig. 5D,E). Cells were grown in the presence of increasing concentrations of AZ1 for 72 h, followed by *in vivo* exposure to propidium iodide (PI) as a marker for dead cells (Fig. 5E) and immunofluorescent staining of the proliferative marker Ki‐67 (Fig. 5D). While the nononcogenic BEAS‐2B^DIF^ showed weak reduction in cell proliferation and mild increase in nuclear PI‐positive cells, BEAS‐2B^ONC^ cells, irrespective of oncogenic driver, significantly reduced cell proliferation in an AZ1 concentration‐dependent fashion (Fig. 5D). Additionally, when BEAS‐2B^ONC^ cells were exposed to AZ1 concentrations reaching or exciding the oncogene corresponding to IC_50_, PI incorporation was significantly enriched (Fig. 5D,E). Surprisingly, BRAFV600E‐transformed BEAS‐2B demonstrated a high degree of sensitivity towards AZ1 (Fig. 5E), as it was previously reported that, in melanoma, loss of USP28 was required to induce oncogenic transformation and resistance.

Overall, our data demonstrated that USP28 is required to maintain tumour cell proliferation and survival *in cellulo*; hence, tumour cells become addicted to USP28.

### 3.6 Inhibition of USP28 via AZ1 ‘resets’ the proteome of oncogenic transduced cells towards a ‘nononcogenic’ state and induces proapoptotic signatures

**Fig. 6:**
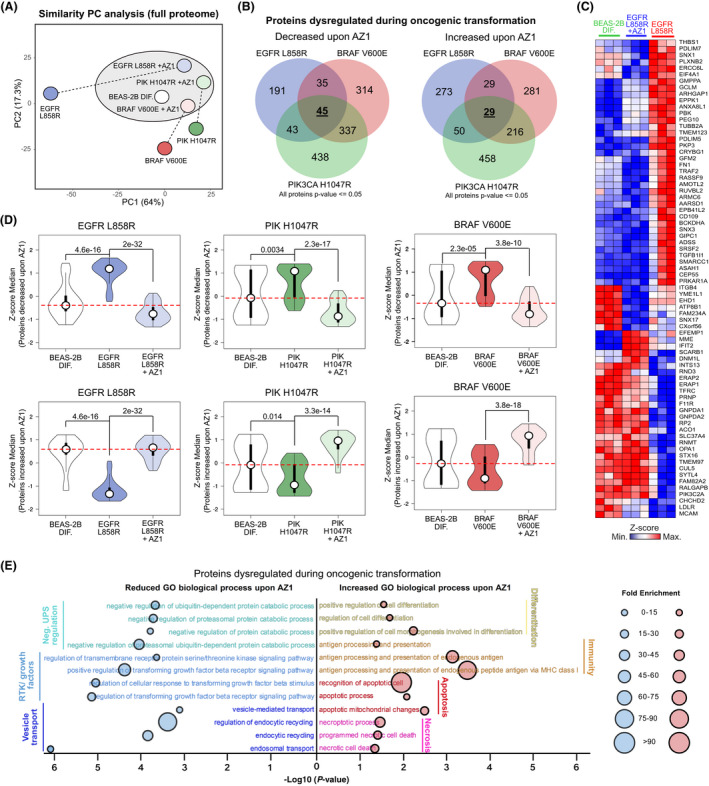
Inhibition of USP28 via AZ1 ‘resets’ the proteome of oncogenic transduced cells towards a ‘nononcogenic’ state and induces proapoptotic signatures. (A) Principal component analysis (PC analysis) of the whole proteome of BEAS‐2B^DIF^ and BEAS‐2B^ONC^ (EGFRL858R, PIK3CA L1047R), treated with either 15 µm AZ1 for 72 h or exposed to control solvent (DMSO). *n* = 3. Analysis generated with ClustVis online tool (https://biit.cs.ut.ee/clustvis). (B) Venn diagram illustrating proteins changed upon exposure of BEAS‐2B^ONC^ (EGFRL858R, PIK3CA L1047R) to 15 µm AZ1 for 72 h. Highlighted are numbers of proteins dysregulated during oncogenic transformation (BEAS‐2B → BEAS‐2B^ONC^) and decreased or increased upon AZ1 exposure. Discreet or common deregulated protein numbers are indicated within the corresponding overlapping graphs. Common, oncogenic driver independent number of deregulated proteins is highlighted in the centre. Analysis of *n* = 3. (C) Heatmap of proteins identified in (B) for BEAS‐2B^DIF^, BEAS‐2B^EGFRL858R^ and BEAS‐2B^EGFRL858R^ treated with 15 µm AZ1 for 72 h. Shown are *n* = 3 experiments and data presented as *Z*‐score values. Red = high *Z*‐score protein abundance, blue = low *Z*‐score protein abundance. (D) Violin plots illustrating changes in BEAS‐2B^DIF^ and BEAS‐2B^ONC^ pre‐ and post‐treatment with 15 µm AZ1 for 72 h for decreased or increased proteins identified in (B). *P*‐values were calculated using the two‐tailed *t*‐test statistical analysis. Red dashed lines reflect the difference between BEAS‐2B^DIF^, BEAS‐2B^Onc^ (EGFRL858R, PIK3CA L1047R and BRAFV600E) and BEAS‐2B^Onc^ upon exposure to 15 µm AZ1 for 72 h. For violin plot, white circles show the medians; box limits indicate the 25th and 75th percentiles as determined by R software; whiskers extend 1.5 times the interquartile range from the 25th and 75th percentiles; polygons represent density estimates of data and extend to extreme values. Violin plot was generated with BoxPlotR. http://shiny.chemgrid.org/boxplotr/. (E) Gene ontology (GO) biological process significantly reduced or increased in BEAS‐2B^ONC^ upon 15 µm AZ1 for 72 h. Horizontal line indicates −log10 of the *P*‐value, and the area of the circular mark reflects the fold enrichment. *P*‐value was calculated using Fisher's exact test using http://www.pantherdb.org. The analysis was performed with the proteins identified in (B). The analysis was performed using the online tool Panther. http://www.pantherdb.org. ONC, oncogenic; RTK, receptor tyrosine kinase. See also Fig. S7.

To gather further insights into how targeting of USP28 via AZ1 affects oncogenic transduced BEAS‐2B cells, we conducted mass spectrometric analysis and compared the proteome of control and oncogenic transduced cells upon exposure to AZ1 (Fig. S7A). The principal component analysis identified that oncogenic transduction resulted in distinct changes of the proteome (Fig. 6A and Fig. S7B). Not only were the proteomes of BEAS‐2B^onc^ different when compared to non‐oncogenic cells, but also the oncogenic driver used established distinct proteomic patterns (Fig. 6A). Upon exposure to AZ1, the proteomes of BEAS‐2B^onc^, however, significantly changed and clustered with the expression of nononcogenic BEAS‐2B (Fig. 6A and Fig. S7C). Analysing the proteome of the three BEAS‐2B^onc^ cell lines post‐AZ1 exposure revealed that a set of proteins dysregulated during oncogenic transformation was commonly affected in an AZ1‐dependent fashion; 45 proteins were decreased and 29 proteins commonly increased in BEAS‐2B^onc^ (Fig. 6B,C and Fig. S7D). Proteins upregulated in EGFRL858R‐transduced BEAS‐2B were repressed upon exposure to AZ1, while proteins decreased during the course of oncogenic transformation enriched upon blockage of USP28 activity (Fig. 6C,D and Fig. S7D). Addition of 15 µm AZ1 for 72 h, however, not only reduced the abundance of proto‐oncogenes, but re‐shaped global protein abundance, closer resembling non‐oncogenic BEAS‐2B (Fig. 6C,D). Inhibition of USP28 via AZ1 in oncogenic transduced BEAS‐2B significantly repressed the abundance of proteins involved in negative regulation of the ubiquitin–proteasome system, and decreased RTK/growth factor signalling and vesicle transport, while oncogenic BEAS‐2B‐upregulated proteins involved in differentiation, immune signalling, apoptosis and necrosis (Fig. 6E).

Hence, acute inhibition of USP28 via AZ1 decreases the amount of proto‐oncogenes, thereby affecting global protein abundance. USP28 is required to accommodate oncogenic transformation and to suppress antiproliferative and pro‐apoptotic signatures.

### 3.7 USP28 inhibition potentiates targeted molecular therapy

**Fig. 7:**
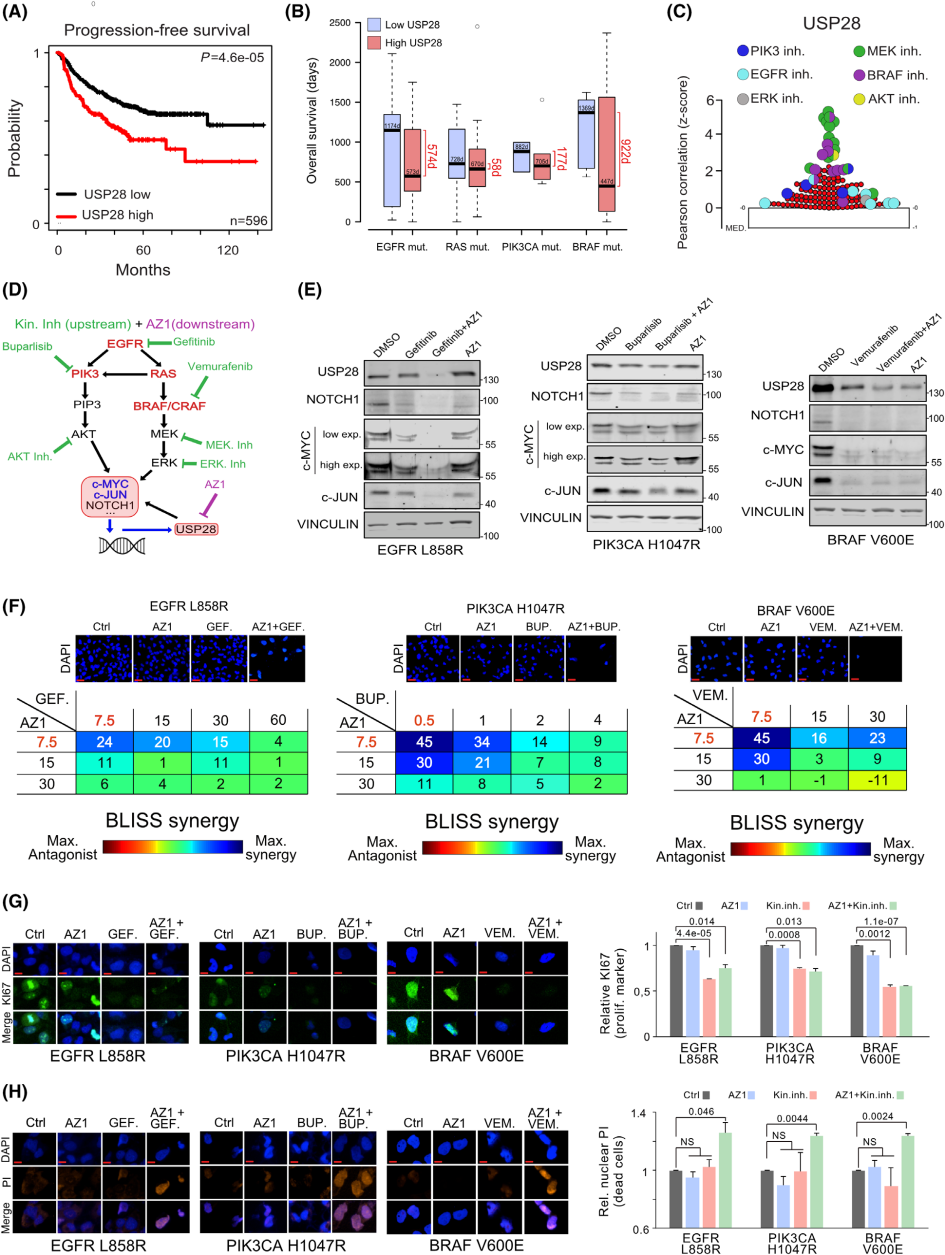
USP28 inhibition potentiates targeted molecular therapy. (A) Kaplan–Meier plot of PFS of NSCLC patients relative to USP28 mRNA expression. *n* = 596 samples, *P *= < 0.0005. Data were generated with the online tool www.kmplot.com. *P*‐values were calculated using the survival log‐rank statistical test. (B) Analysis of publicly available data sets analysing USP28 mRNA expression and overall survival (OS) in patients with mutations in the oncogenic driver EGFR (*n* = 33), RAS (*n* = 41), PIK3CA (*n* = 10) or BRAF (*n* = 17). Samples were divided into two groups based on USP28 mRNA expression: high USP28 (higher than the median USP28 expression) and low USP28 (lower than the median USP28 expression). Survival days were determined for both groups. Data were obtained from the online tool https://xena.ucsc.edu/. In box plots, the centre line reflects the median and the upper and lower box limits indicate the first and third quartiles. Whiskers extend 1.5× the IQR. Plot was generated using the online tool http://shiny.chemgrid.org/boxplotr/. (C) Pearson's correlation between sensitivity of EGFR‐PI3K‐MAPK inhibitors and USP28 expression in human NSCLC cell lines. Data were represented using *Z*‐Scores, and the graphic was generated with the online tool Cancer Therapeutics Response Portal V2 (https://portals.broadinstitute.org/ctrp/.v2.1). In the online tool, 531 compounds were analysed (*n* = 531). For the graphical plot, the lower line reflects the median and the upper box limit indicates the first quartile. Whiskers extend 1.5× the IQR. (D) Schematic representation of EGFR‐PI3K‐MAPK pathway analysed in this study and potential pathway interference opportunities by EGFR‐PI3K‐MAPK and AZ1 inhibitors. Green = EGFR‐PI3K‐MAPK inhibitors. Violet = AZ1. (E) Immunoblots against USP28, NOTCH1, c‐MYC and c‐JUN of BEAS‐2B^ONC^ (EGFRL858R, PIK3CA L1047R and BRAFV600E) cultured in the presence of either control solvent (DMSO), pathway‐specific inhibitors (EGFR: 20 µm gefitinib; PIK3CA: 1 µm buparlisib; and BRAF: 20 µm vemurafenib), 15 µm AZ1 or a combination thereof for 24 h. Vinculin serves as loading control. *n* = 3. (F) BLISS synergism score of BEAS‐2B^ONC^ (EGFRL858R, PIK3CA L1047R and BRAFV600E) cultured in the presence of either control solvent (DMSO), pathway‐specific inhibitors (EGFR: gefitinib; PIK3CA: buparlisib; and BRAF: vemurafenib), AZ1 and combination thereof for 72 h at indicated concentrations. Shown are representative DAPI images of cells 72 h postculture in the presence of DMSO, single treatment with 7.5 µm AZ1, 7.5 µm gefitinib, 0.5 µm buparlisib, 7.5 µm VEMURAFENIB or combination of AZ1 with the respective personalized molecular therapy for 72 h. Synergism was calculated by cell quantification of 30–45 20× fields from three independent wells in control (DMSO) and the indicated treated conditions. combenefit software was used to calculate synergism. *n* = 3. Scale bar = 15 µm. (G) Immunofluorescence of Ki‐67 expression in oncogenic transduced BEAS‐2B^ONC^ (EGFRL858R, PIK3CA L1047R and BRAFV600E), cultured in the presence of DMSO, single treatment with 7.5 µm AZ1, 7.5 µm gefitinib, 0.5 µm buparlisib, 7.5 µm vemurafenib or combination of AZ1 with the respective pathway inhibitor for 72 h, to assess effects in cell proliferation. Shown are representative cells. Quantification of Ki‐67 expression in 30–45 20× fields from different wells in control (DMSO) and treated conditions. DAPI served as nuclear marker. *n* = 3. Shown are mean values and standard deviation. *P*‐values were calculated using the two‐tailed *t*‐test statistical analysis. Scale bar = 5 µm. (H) Immunofluorescence of propidium iodide (PI) *in vivo* incorporation of oncogenic transduced BEAS‐2B^ONC^ (EGFRL858R, PIK3CA L1047R and BRAFV600E), cultured in the presence of DMSO, single treatment with 7.5 µm AZ1, 7.5 µm gefitinib, 0.5 µm buparlisib, 7.5 µm vemurafenib or combination of AZ1 with the respective pathway inhibitor for 72 h, to assess effects in cell proliferation. Shown are representative cells. Quantification of PI‐positive cells in 30–45 20× fields from different wells in control (DMSO) and treated conditions. DAPI served as nuclear marker. Shown are mean values and standard deviation. *P*‐values were calculated using the two‐tailed *t*‐test statistical analysis. *n* = 3. Scale bar = 5 µm. BUP, buparlisib; Ctrl, control; Exp., exposure; GEF, gefitinib; Inh., inhibitor; ONC, oncogenic; VEM, vemurafenib. See also Fig. S8.

To investigate whether USP28 is a putative prognostic marker for NSCLC, we analysed publicly available data sets and observed that USP28 expression significantly correlates with progression‐free survival (PFS, *P* = 4.6e‐05, Fig. 7A and Fig. S8A). In the post progression survival cohort, elevated expression of USP28 directly correlated with shortened survival (overall and Stage I, S8A and S8B). These data suggest that USP28 levels strongly determine the survival of the NSCLC patients in response to therapy.

Since NSCLC is a genetically very heterogeneous tumour type, next, we wondered whether patient survival depended on the combination of oncogenic driver and the expression of USP28 (Fig. 7B and Fig. S8C,D). Indeed, the analysis of public available data leads to the suggestion that, irrespective of oncogenic driver, an increased expression of USP28 significantly shortens survival for tumours driven by mutations in the oncogenes EGFR (∆574 days), PIK3CA (∆177 days) or BRAF (∆922 days), while in tumours driven by mutations in genes of the RAS family, USP28 had very little effect on patient survival (∆58 days; Fig. 7B and Fig. S8D). Furthermore, the mutational status of the tumour suppressor TRP53 presented no prognostic value with regard to USP28 expression in patients diagnosed with NSCLC (Fig. S8D). These data suggest that USP28 is a suitable prognostic marker and oncogene in NSCLC.

As cancer panel sequencing is implemented in the clinics and pathway‐specific inhibitors are available, we were wondering whether disruption of the oncogenic pathways would directly affect USP28 and hence the abundance of its downstream effectors. Analysis of publicly available data regarding putative drug sensitivity of tumour cells in direct correlation to USP28 expression scored the PIK3, EGFR and MAPK pathway as top hits (Fig. 7C), which directly confirmed our experimental data regarding USP28 expression relative to oncogenic drivers. Since several potent pathway inhibitors are readily available (Fig. 7D), we wondered whether targeted therapy would synergize with targeted inhibition of USP28 via AZ1. To this end, we exposed our BEAS‐2B^ONC^ cell lines to either a selective inhibitor (EGFRL858R = gefitinib; PIK3CAH1047R = buparlisib; and BRAFV600E = vemurafenib), AZ1, or a combination thereof for 24 h, followed by immunoblotting against USP28 and its substrates (Fig. 7E). Selective pathway interference was evaluated by immunoblotting (Fig. S7E). Monotherapy via selective pathway inhibitors affected downstream signalling cascades, reduced the abundance of USP28, led to a reduction in the protein levels of NOTCH1, c‐MYC and c‐JUN and reduced cell viability (Fig. 7E). Similar effects were observed by administering AZ1; treated cells showed a reduction in USP28 abundance, along with reduced protein levels of its substrates and decreased viability (Fig. 7E). Combinatorial treatment, however, significantly reduced the amount of USP28 and diminished the abundance of NOTCH1, c‐MYC and c‐JUN (Fig. 7E). Not only does cotreatment with AZ1 sensitize BEAS‐2B^ONC^ cells to targeted therapy, but also it synergizes with gefitinib, buparlisib and vemurafenib, as seen by viability assays in BEAS‐2B^ONC^ cells (Fig. 7F and Fig. S8F). The synergistic effect of AZ1 and targeted therapy does not only stem from impairment of tumour cell proliferation but also leads to onset of cell death, as seen by Ki‐67 expression and PI incorporation experiments of BEAS‐2B^ONC^ cells cultivated for 72 h in the presence of the single compounds or combinations thereof at synergistic concentrations (AZ1: 7.5 µm, gefitinib: 7.5 µm; buparlisib: 0.5 µm; and vemurafenib: 7.5 µm; Fig. 7G,H). Here, exposure to 7.5 µm AZ1 did not affect proliferation nor induced cell death (Fig. 7G,H and Fig. S8F). While monotherapy with targeted inhibitors led to a reduction in proliferation, it did not result in cell death, as seen by loss of Ki‐67 expression and the absence of PI incorporation (Fig. 7G,H). Extended coculture of engineered oncogenic BEAS‐2B for up to 144 h confirmed that cotreatment of kinase‐specific inhibitors with AZ1 as reduced concentrations (3.75 µm AZ1, 3.75 µm gefitinib, 0.25 µm buparlisib and 3.75 µm vemurafenib) does provide a long‐term antiproliferative effect (Fig. S8F). The combinatorial treatment, however, resulted in a significant increase in PI‐positive cells, while maintaining a proliferative block (Fig. 7G,H).

To investigate whether the combinatorial treatment of USP28 inhibition and targeted kinase inhibitor therapy is also suitable for genetically complex cancer cells, next, we co‐cultured the BRAF mutant human cancer cell lines A375 and HT‐29, the EGFR mutant cell lines H1650 and A431, and the PIK3CA mutant lines HT‐29 and CaSki in the presence of the respective inhibitors at increasing concentrations for 72 h (Fig. S8G). In line with our observations derived in genetically tailored cellular systems (BEAS‐2B), we could observe a synergistic effect of various extents in HT‐29, H1650 and A431, respectively, while A375 and CaSki showed no synergistic nor additive effect.

USP28 therefore functions as a signal amplifier downstream of various oncogenic signalling cascades and is required to allow oncogenic transformation. Its inhibition potentiates targeted therapy by impairing the protein stability of oncogenic transcription factors, required to maintain tumour cell proliferation, and presents a promising target for drug development.

## 4 Discussion

Oncogenic transformation of somatic cells is a multistage process frequently starting with the inactivation of tumour suppressors and subsequent gain of activating mutations in oncogenic drivers, such as members of the PI3K or MAPK family. These changes result in the increased abundance of proto‐oncogenes, such as c‐MYC [44], JUN [45] or NOTCH, driving cell proliferation, dedifferentiation, metabolic changes, DNA damage control, immune evasion and proteostatic stress management, the ‘hallmarks of cancer’ [34].

**Fig. 8:**
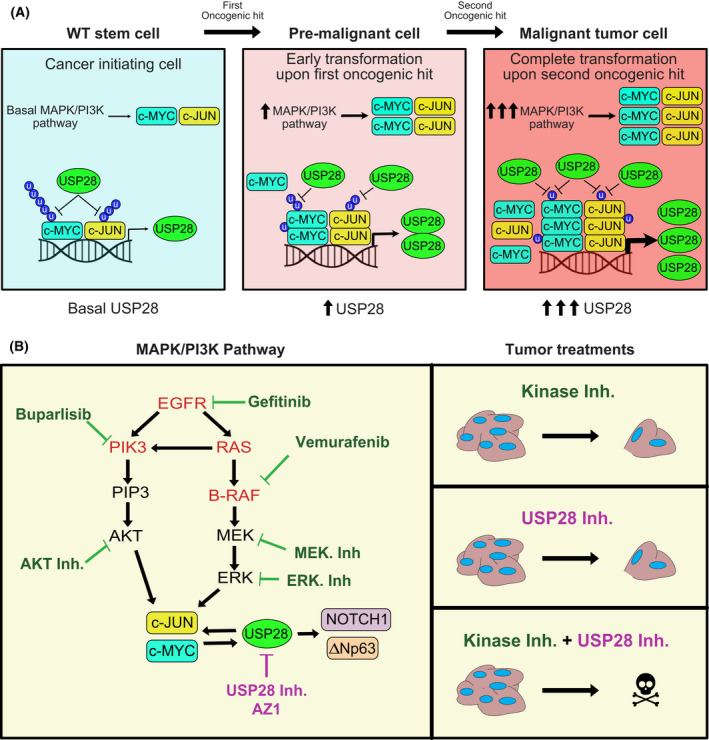
USP28 enables oncogenic reprograming of respiratory cells during early transformation and its inhibition potentiates targeted molecular therapy. (A) Schematic representation of the mechanism presented in this manuscript. Oncogenic transformation via EGFR‐PI3K‐MAPK pathway increases USP28 transcription via c‐MYC/ c‐JUN. USP28 stabilizes the oncoproteins c‐MYC/ c‐JUN stablishing a direct feedback loop. (B) Schematic representation of the synergy between EGFR‐PI3K‐MAPK targeted molecular therapies and AZ1 presented in this manuscript.

Cells undergoing transformation partially counter this intrinsic stress by readjusting the UPS. USP28 is involved in the control of a plethora of biological processes. It is involved in the regulation of cell proliferation and differentiation via its ability to regulate the abundance of the proto‐oncogenes, such as c‐MYC, c‐JUN or NOTCH (Fig. 8A) [40]. It is involved in transcriptional control via regulating the abundance of the histone modifier LSD1/KDM1A [46], and it is part of the DNA damage machinery, where it interacts with ATM [47], CLSPN [48], or TP53BP1 [49].

Overall, USP28 functions as a proto‐oncogene and contributes to establishing the hallmarks of cancer in cells undergoing oncogenic transformation, at least in lung [21, 50, 51, 52]. Here, USP28 is expressed in the stem cell niche and elevated abundance detected in ‘cells of origin’. Its upregulation is an early event, as already low‐grade tumours showed enhanced abundance for this particular DUB, and its expression coincided with shortened overall survival. In a cellular multistage transformation model, we were able to recapitulate the stepwise increase in USP28 abundance and enhanced activity, which occurred independent of the oncogenic driver present. USP28 is a transcriptional target of its own substrates [40] and, via a feed‐forward loop, increased in cancer compared with normal cells. Irrespective of oncogenic driver, interference with USP28 abundance or activity suppressed tumour cell growth and survival of transformed lung cells *in vitro* and *in vivo*. Inhibition of USP28 via the small‐molecule inhibitor AZ1 restored the proteome of oncogenic transformed BEAS‐2B cells towards a premalignant state, demonstrating that interference with USP28 abundance and activity has a far‐reaching biological impact. USP28 is a downstream target of RTK signalling cascades and required to establish oncogenic transformation by its ability to control the abundance of proto‐oncogenes. The inhibition of the DUB not only repressed tumour cell growth but led to a proapoptotic phenotype, predominantly in oncogenic transformed cells, while control cells were not affected. Here, the cell cycle was affected, leading to a reduction in proliferation.

This is in stark contrast to two recent reports. In a small cohort of melanoma patients, where tumours are predominantly driven by mutant BRAF(V600E), USP28 is genetically lost [53]. These USP28 mutant patients present enhanced MAPK signalling via hyperstabilization of RAF family members and resistance to BRAF inhibitors. Here, loss of USP28 presents a negative survival marker. The second study identified that, in melanoma cells, USP28 is cleaved by caspase‐8 to overcome G2/M cell cycle arrest in a TP53‐dependent fashion [54]. Loss of USP28 in tumour cells is favoured as it results in TP53 protein destabilization, thereby establishing a switch of cell fate, from apoptosis towards mitosis. Hence, USP28 functions as a tumour suppressor in melanoma [55]. In line with these reports, we could not detect a correlation between USP28 and BRAF expression in human skin cancer samples. This could be indicating that this tumour entity indeed does not rely on the DUB, and hence, alternative mechanisms deviating from our observation in lung and SCC are possible.

In lung, and as previously reported for SCC [21], targeting USP28 presents a suitable lever for therapeutic engagement, as one could propose that at least in lung, tumour cells become addicted to USP28. In line with this hypothesis, we indeed did observe that loss of USP28 reduced oncogenic cell proliferation and its genetic loss impaired tumour onset *in vivo*. The inhibition of USP28, via a small‐molecule inhibitor AZ1, induced proapoptotic signalling in cells expressing potent oncogenic driver mutants. In lung, the expression of USP28 directly correlated with shortened patient survival, irrespective of oncogenic driver. As USP28 functions as an ‘amplifier’ downstream of RTKs, its inhibition cooperated with personalized targeted therapy against specific oncogenic driver mutations (Fig. 8B).

## 5 Conclusion

Overall, our data suggest that targeting USP28 protein abundance and activity already at an early stage therefore is a promising strategy for the treatment of lung tumours in combination with personalized targeted therapy.

## Conflict of interest

The authors declare no conflict of interest.

## Author contributions

CP‐G and MED conceptualized the study. CP‐G, MRe, FB, NP, CF and AS contributed to *in vitro* methodology. OH, MRo and MED contributed to *in vivo* methodology. SB, ID and CM contributed to proteomics methodology. CS‐V contributed to Operetta system methodology. CP‐G, MRe, SB, ID and CM performed bioinformatic formal analysis. MACC, MRo and MED performed pathologic formal analysis. CP‐G, OH, MRe, FB, NP, CF, MRo and MED investigated the study. MACC, MRo, AB, ID, CM and MED provided resources. MED wrote the original draft, supervised the study and acquired funding. CP‐G, OH, MRe, MRo, AB, ID and MED wrote, reviewed and edited the manuscript.

### Peer review

The peer review history for this article is available at https://publons.com/publon/10.1002/1878‐0261.13217.

## Supporting information


**Fig. S1.** USP28 is upregulated irrespective of lung tumour subtype.
**Fig. S2.** USP28 is required to establish oncogenic transformation *in vivo*.
**Fig. S3.** USP28 is upregulated during early stages of transformation in the human cell line model system, BEAS‐2B.
**Fig. S4.** Malignant transformation of BEAS‐2B^DIF^ via EGFR‐PI3K‐MAP.
**Fig. S5.** Oncogenic transformation via EGFR‐PI3K‐MAPK pathway upregulate USP28.
**Fig. S6.** Malignant transformation renders tumour cells dependent on USP28.
**Fig. S7.** Inhibition of USP28 via AZ1 ‘resets’ the proteome of oncogenic transduced cells towards a ‘nononcogenic’ state and induces proapoptotic signatures.
**Fig. S8.** USP28 inhibition potentiates targeted molecular therapy.

**Table S1.** Consumables and resources.
