# Supplementary material for: USP28 enables oncogenic transformation of respiratory cells, and its inhibition potentiates molecular therapy targeting mutant EGFR, BRAF and PI3K
Source: Mol Oncol. 2022 Apr 30;16(17):3082–106. doi: 10.1002/1878-0261.13217 (PMC9441007; doi:10.1002/1878-0261.13217)

Supplementary Figure Legends

Figure S1: ***USP28 is upregulated irrespective of lung tumour subtype***

A) Publicly available data of relative mRNA expression of USP28 in human NSCLC Adenocarcinoma samples, separated by subclass. Shown are non-transformed and tumour samples. N = normal lung tissue, T = tumour. n indicates number of individual samples. Generated with the open-source tool www.gepia2.cancer-pku.cn. p-values were calculated using two-tailed T-test statistical analysis. p<0.005 = ***. In box plots, the centre line reflects the median and the upper and lower box limits indicate the first and third quartiles. Whiskers extend 1.5× the IQR.
B) Publicly available data of relative mRNA expression of USP28 in human NSCLC Squamous cell carcinoma samples, separated by subclass. Shown are non-transformed and tumour samples. N = normal lung tissue, T = tumour. n indicates number of individual samples. Generated with the open-source tool www.gepia2.cancer-pku.cn. p-values were calculated using two-tailed T-test statistical analysis. p<0.0005 = ****. In box plots, the centre line reflects the median and the upper and lower box limits indicate the first and third quartiles. Whiskers extend 1.5× the IQR.

Figure S2: ***USP28 is required to establish oncogenic transformation in vivo.***

A) Overview of genetic alterations within the ERBB, RAS, PI3K and RAF oncogenic families in NSCLC (SCC and ADC). Shown are total percent of genetic alterations within the respective pathway and individual pathway members. N = 501 SCC and n = 515 ADC samples. Data generated with the online tools www.cbioportal.org.
B) *Schematic representation of in vivo* CRISPR mediated gene editing to induce deletion of *Trp53* and mutation of *Braf* to *Braf*^{V600E} (PB) upon intratracheal instillation of AAV virions, packaged with the pleotropic capsid 2/DJ, into *C57Bl6/J-Rosa26*$^{Sor\text{-}CAGG\text{-}Cas9\text{-}IRES\text{-}eGFP}$ mice. Animals were sacrificed 4 weeks post infection. n= 3.
C) Representative images of immuno-histochemical staining against endogenous Usp28, Nkx2-1/Ttf-1 and the proliferative marker Pcna in murine lungs infected with either control virus (WT) or PB. Shown a representative lung, with a low and high magnification of individual areas. Animals were analysed 4 weeks' post-infection n=3

Figure S3: USP28 is upregulated during early stages of *transformation in the human cell line model system, BEAS-2B.*

A) Representative brightfield images of BEAS-2B cells propagated as either undifferentiated, stem cell like cells, or upon induction of trans-differentiation, caused by addition of 10%FCS to the growth medium for 3 days.
B) Representative immunofluorescent images against endogenous USP28 (green) of BEAS-2B cells propagated as either undifferentiated, stem cell like cells, or post

serum-induced trans-differentiation, caused by addition of 10%FCS to the growth medium for 3 days. DAPI served as nuclear marker. n= 3.

C) Representative enlarged brightfield images of experiment shown in Figure 3E.

Figure S4: ***Malignant transformation of BEAS-2B^{DIF} via EGFR-PI3K-MAP.***

A) mRNA expression correlation data between USP28 and RAS family members H-RAS, K-RAS and NRAS in NSCLC ADC and SCCC, obtained by public available data sets (www.gepia2.cancer-pku.cn/). Shown is the Spearman correlation efficiency (R).

B) Graphical visualization of the Spearman´s correlation between USP28 and KRAS or NRAS in ADC tumours. Correlation and p-value calculated were calculating using the online tool GEPIA www.gepia2.cancer-pku.cn/ .

C) Schematic overviews of commonly occurring mutations in NSCLC driver oncogenes EGFR, HRAS, BRAF and PIK3CA. Shown are missense (green), truncating mutations (black), in frame mutations (brown) and other genetic alterations (violet). Data was generated using the online tool www.cbioportal.org.

D) Immunoblot against EGFR, HRAS, BRAF and PIK3CA oncogenic drivers in BEAS-2B^{DIF} or BEAS-2B^{DIF} upon retroviral transduction to express the indicated oncogenes EGFR (wild type (WT) and L858R), HRAS (G12D), BRAF (V600E) and PIK3CA (wild type (WT), E545K and H1047R), respectively. ACTIN served as loading control. Representative immunoblot of n=3.

E) RT-PCR of EGFR, HRAS, BRAF and PIK3CA oncogenic drivers in BEAS-2B^{DIF} or BEAS-2B^{DIF} upon retroviral transduction to express the indicated oncogenes EGFR (wild type (WT) and L858R), HRAS (G12D), BRAF (V600E) and PIK3CA (wild type (WT), E545K and H1047R), respectively. Shown are log2 fold change expression data, relative to ACTIN and normalized to the respective expression in BEAS-2B^{DIF}. Shown are mean values and standard deviation of n=3

Figure S5: ***Oncogenic transformation via EGFR-PI3K-MAPK pathway upregulate USP28.***

A) Schematic model. c-JUN and c-MYC up-regulate transcription of USP28 (Serra et al. 2014; Diefenbacher et al. 2014).

B) Immunoblot against USP28, c-MYC, c-JUN in BEAS-2B^{DIF} or BEAS-2B^{DIF} upon transfection of cDNA encoding c-JUN and c-MYC. VINCULIN served as loading control. Representative immunoblot of n=3.

C) Immunoblot against endogenous USP28 and its substrates NOTCH1, c-MYC and c-JUN of BEAS-2B^{UD}, BEAS-2B^{DIF} and BEAS-2B^{DIF} upon retroviral transduction to express the indicated oncogenes EGFR (L858R), HRAS (G12D), BRAF (V600E) and PIK3CA (wild type (WT) and H1047R), respectively. VINCULIN or ACTIN served as loading control. Representative immunoblot of n=3.

D) Overview of the oncogenic pathways and recurring oncogenic drivers involved in the regulation of USP28 expression and abundance. Oncogenic transformation upon activation of EGFR-PI3K-MAPK pathway up-regulates transcription of USP28 via c-MYC/ c-JUN.

E) Pan-Cancer mRNA expression correlation data between USP28 and AKT2 or BRAF, obtained by public available data sets (Depmap.org). Pearson´s correlation between USP28 and AKT2 or BRAF mRNA levels in cancer cell lines from different tumor entities. For graphical visualization, linear regression was included and every dot represent a different cell line. The table indicates the next parameters for different tumor entities: Pearson correlation, slope and p-value for the individual tumour types, relative to AKT2-USP28 and BRAF-USP28. The parameters were calculated by the online tool https://depmap.org/.

Figure S6: ***Malignant transformation renders tumour cells dependent on USP28.***

A) Immunofluorescence of USP28 endogenous expression in A549 cells. A549 is a KRAS mutated cell line (KRASG12S). ACTIN and DAPI as control markers.
B) Immunoblot showing protein abundance of USP28, c-MYC and PCNA in A549 upon lentiviral transduction with either a control or two individual constitutive shRNA targeting USP28. ACTIN served as loading control.
C) For growth analysis, A549 cells from B) were seeded at equal cell density and counted at day 1, day 3 and day 5. p-values were calculated using two-tailed T-test statistical analysis. Shown are mean values and standard deviation of n=3.
D) BEAS-2B^{ONC} (EGFRL858R, PIK3CA L1047R and BRAFV600E) cultured in the presence of either control solvent (DMSO) or AZ1 for 72 hours at indicated concentrations. Shown are representative DAPI Images of cells 72 hours post culture in the presence of DMSO or AZ1. Calculation of GI50 was performed upon cell quantification of 30-45 20x fields from independent wells in control (DMSO) and AZ1 conditions.

Figure S7: ***Inhibition of USP28 via AZ1 'resets' the proteome of oncogenic transduced cells towards a 'non-oncogenic' state and induces pro-apoptotic signatures***

A) Schematic model explaining the proteomics experiments and analysis performed in this manuscript.
B) Volcano plots comparing protein abundances in BEAS-2B^{DIF} and BEAS-2B^{ONC} (EGFRL858R, PIK3CA L1047R and BRAFV600E).
C) Volcano plots comparing protein abundances in BEAS-2B^{ONC} (EGFRL858R, PIK3CA L1047R and BRAFV600E) upon exposure to DMSO or 15 µM AZ1 for 72 hours AZ1.
D) Heatmap of proteins identified in Figure 6B for BEAS-2B^{DIF}, BEAS-2B^{ONC} (PIK3CA L1047R or BRAFV600E) and BEAS-2B^{ONC} (PIK3CA L1047R or BRAFV600E) treated with 15 µM AZ1 for 72 hours. Shown are n=3 experiments and data presented as Z score values per row. Red= high Z-score, blue = low Z-score protein abundance.

Figure S8: ***USP28 inhibition potentiates targeted molecular therapy***

A) Schematic model explaining the concept of overall survival (OS), progression free survival/disease (PFS) and post-progression survival (PPS).
B) Kaplan-Meier plots of NSCLC patient post-progression survival (PPS), relative to USP28 expression for total number of patients, patients at Stage 1 and patients at Stage 2. Data was generated using the online tool www.kmplot.com.
C) Analysis of publicly available datasets of USP28 mRNA expression and Overall Survival (OS) in NSCLC. Samples were divided in two groups based on USP28 mRNA expression: High USP28 (higher than the median USP28 expression) and Low USP28 (lower than the median USP28 expression). Survival days was determined for both groups. Data was obtained from the online tool https://xena.ucsc.edu/. In box plots, the centre line reflects the median and the upper and lower box limits indicate the first and third quartiles. Whiskers extend 1.5× the IQR.
D) Analysis of publicly available datasets of overall patient survival with regard to either EGFR, KRAS, PIK3CA, BRAF or TP53 wild type or mutant status, stratified against low and high USP28 mRNA expression in NSCLC. N=1047 analysed samples. Samples were divided in two groups based on USP28 mRNA expression: High USP28 (higher than the median USP28 expression) and Low USP28 (lower than the median USP28 expression). Differential survival days were determined for WT and mutant groups, respectively. Data was obtained from the online tool https://xena.ucsc.edu/.
E) Immunoblot showing protein abundance of p-EGFR, p-AKT, P-ERK and ERK in BEAS-2B^{ONC} (EGFRL858R, PIK3CA L1047R and BRAFV600E) upon exposure to DMSO or 20 µM Gefitinib/ 20 µM Vemurafenib/ 1 µM Buparlisib for 72 hours. VINCULIN serves as loading control. n=3
F) BEAS-2B^{ONC} (EGFRL858R, PIK3CA L1047R and BRAFV600E) cultured in the presence of either control solvent (DMSO), 7.5 µM AZ1 or 10 µM Gefitinib/ 10 µM Vemurafenib/ 0.25 µM Buparlisib for 144 hours. At endpoint, *In Cell* western blot against endogenous LaminA/C was conducted to assess cell viability. Relative fluorescent intensity was measured for 4 independent wells. Bar graphs represent the mean values and standard error of the mean. p-values were calculated using two-tailed T-test statistical analysis (Graphpad).
G) Representative Images of In Cell Western Blot and BLISS synergism score of various human cancer cell lines, exposed to either AZ1 or the corresponding targeted kinase inhibitors Vemurafenib, Gefitinib or Buparlisib. A375 and HT-29 cells were used as representative cell lines harbouring BRAFV600E mutations, H1650 and A431 as cell lines with EGFR mutations and HT-29 and CaSki with PIK3CA mutations. All cell lines were cultured in the presence of either control solvent (DMSO), pathway specific inhibitors (EGFR: Gefitinib; PIK3CA: Buparsilib; BRAF: Vemurafenib), AZ1 and combination thereof for 72 hours at indicated concentrations (in µM). At endpoint, *In Cell* western blot against endogenous LaminA/C was conducted to assess cell viability. Relative fluorescent intensity was measured for 4 independent wells and used to calculate the Synergism score.

A

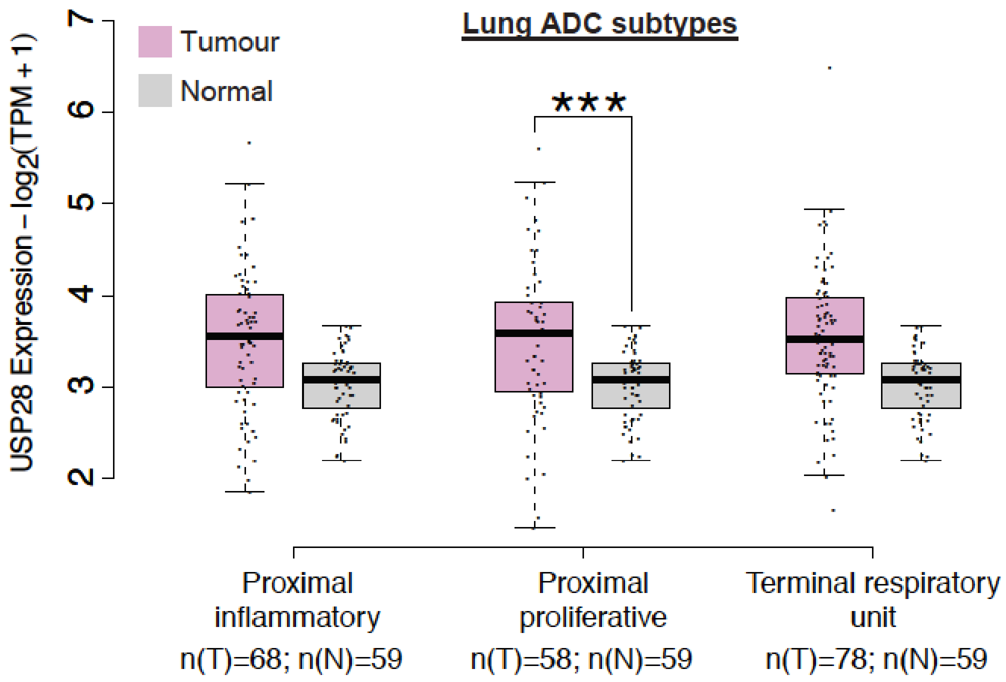
Lung ADC subtypes
Tumour
Normal
USP28 Expression – log2(TPM + 1)
7
6
5
4
3
2
***
Proximal inflammatory
n(T)=68; n(N)=59
Proximal proliferative
n(T)=58; n(N)=59
Terminal respiratory unit
n(T)=78; n(N)=59

B

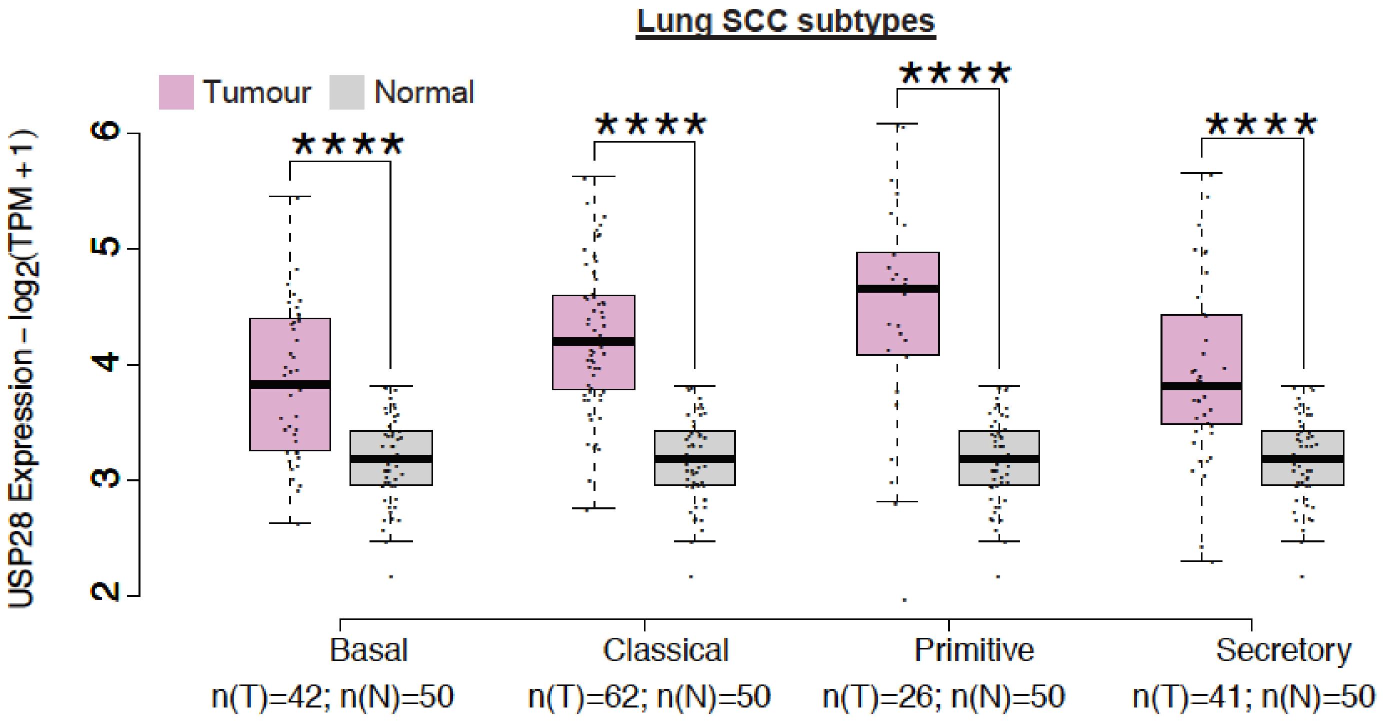
Lung SCC subtypes
Tumour
Normal
USP28 Expression – log2(TPM + 1)
6
5
4
3
2
****
****
****
****
Basal
n(T)=42; n(N)=50
Classical
n(T)=62; n(N)=50
Primitive
n(T)=26; n(N)=50
Secretory
n(T)=41; n(N)=50

A

	ErbB Family		RAS Family		PIK3 class1 Family		RAF Family	
N=501	SCC (43% altered)		SCC (45% altered)		SCC (85% altered)		SCC (63% altered)	
	EGFR	13%	HRAS	8%	PIK3CA	77%	A-RAF	19%
	HER1	17%	**KRAS**	25%	PIK3CB	35%	**B-RAF**	28%
	HER2	13%	NRAS	22%	PIK3CD	3%	C-RAF	39%
	HER3	13%			PIK3CG	11%		
N=515	ADC (59% altered)		ADC (47% altered)		ADC (50% altered)		ADC (51% altered)	
	EGFR	28%	HRAS	8%	PIK3CA	29%	A-RAF	14%
	HER1	26%	**KRAS**	27%	PIK3CB	18%	**B-RAF**	25%
	HER2	20%	NRAS	22%	PIK3CD	12%	C-RAF	28%
	HER3	9%			PIK3CG	13%	C-RAF= Down-reg.	

B

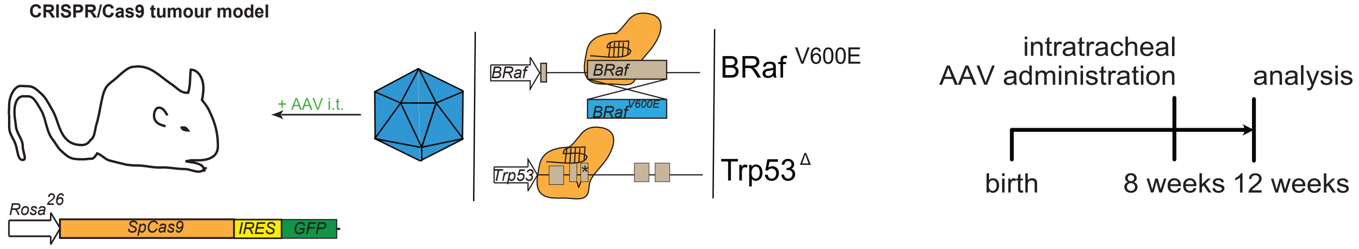

C

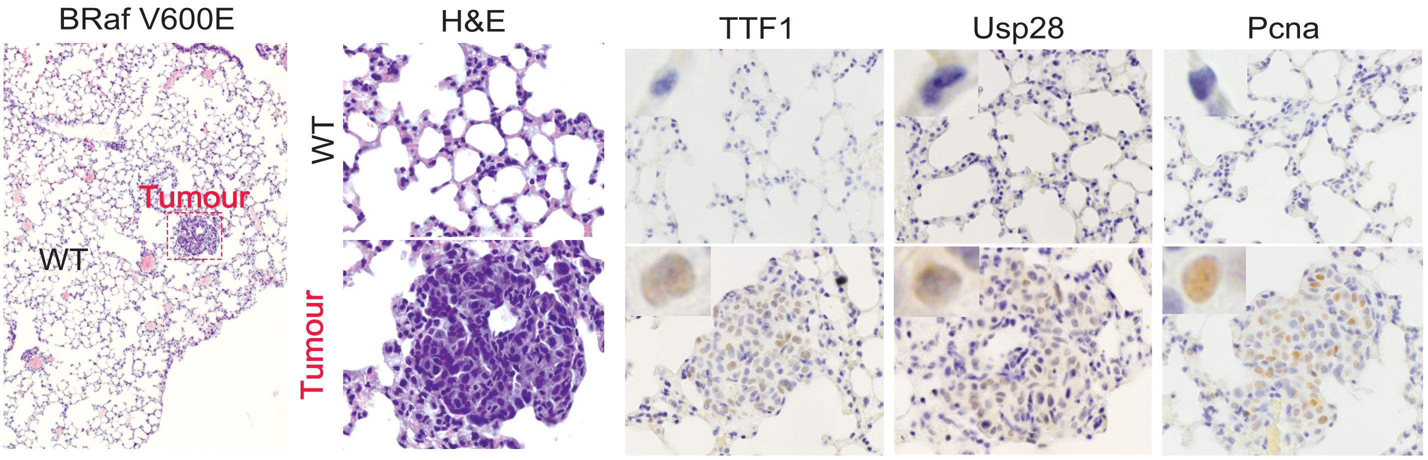

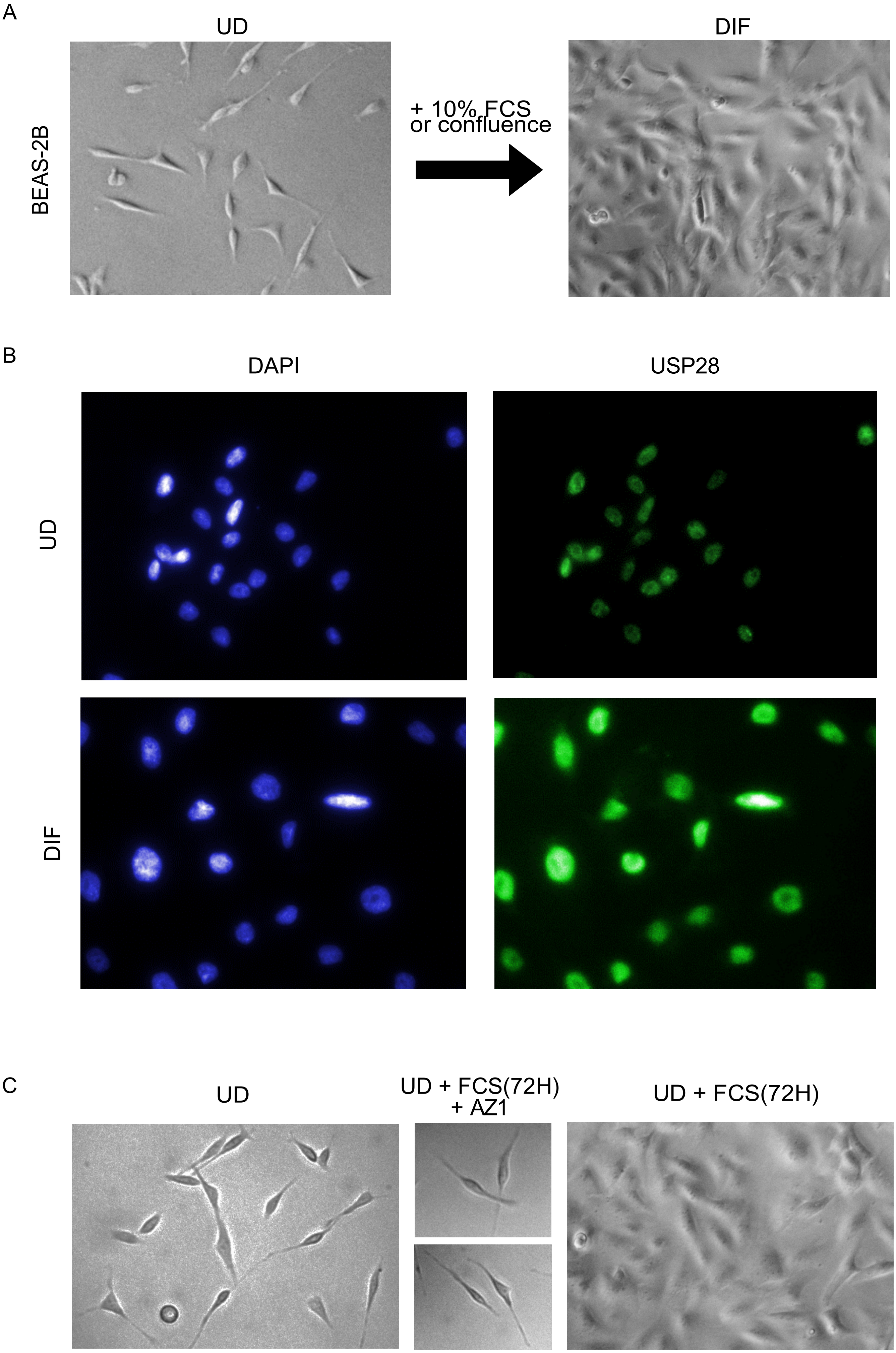
A
UD
DIF
BEAS-2B
+ 10% FCS
or confluence
B
DAPI
USP28
UD
DIF
C
UD
UD + FCS(72H)
+ AZ1
UD + FCS(72H)

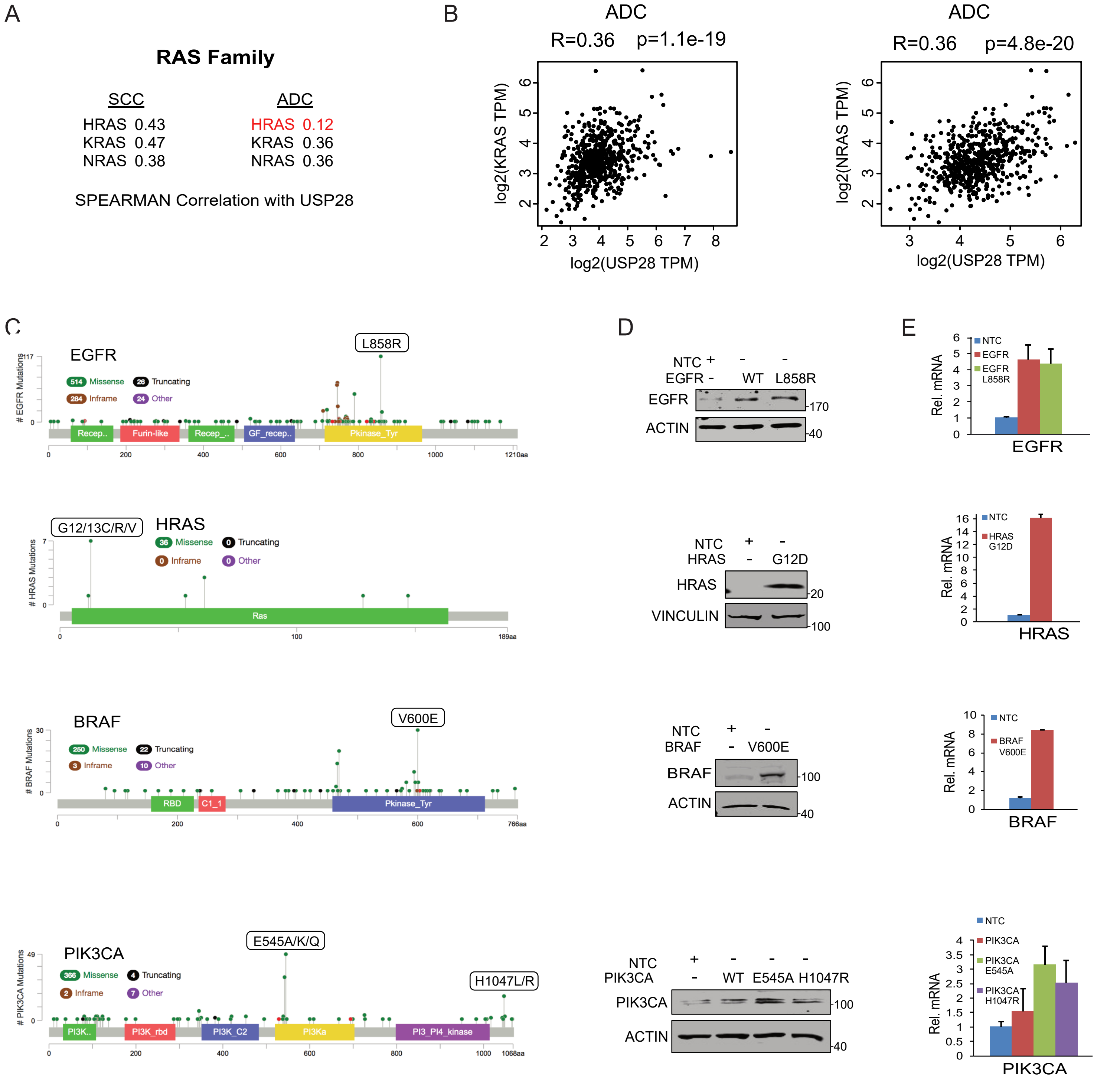
A
RAS Family
SCC
HRAS 0.43
KRAS 0.47
NRAS 0.38
ADC
HRAS 0.12
KRAS 0.36
NRAS 0.36
SPEARMAN Correlation with USP28
B
ADC
R=0.36 p=1.1e-19
log2(KRAS TPM)
log2(USP28 TPM)
ADC
R=0.36 p=4.8e-20
log2(NRAS TPM)
log2(USP28 TPM)
C
EGFR
L858R
514 Missense
26 Truncating
284 Inframe
24 Other
EGFR Mutations
Recep..
Furin-like
Recep_..
GF_recep..
Pkinase_Tyr
1210aa
HRAS
G12/13C/R/V
36 Missense
0 Truncating
0 Inframe
0 Other
HRAS Mutations
Ras
189aa
BRAF
V600E
250 Missense
22 Truncating
3 Inframe
10 Other
BRAF Mutations
RBD
C1_1
Pkinase_Tyr
766aa
PIK3CA
E545A/K/Q
H1047L/R
366 Missense
4 Truncating
2 Inframe
7 Other
PIK3CA Mutations
PI3K..
PI3K_rbd
PI3K_C2
PI3Ka
PI3_PI4_kinase
1068aa
D
NTC + - -
EGFR - WT L858R
EGFR
ACTIN
170
40
NTC + -
HRAS - G12D
HRAS
VINCULIN
20
100
NTC + -
BRAF - V600E
BRAF
ACTIN
100
40
NTC + - - -
PIK3CA - WT E545A H1047R
PIK3CA
ACTIN
100
40
E
NTC
EGFR
EGFR L858R
Rel. mRNA
EGFR
NTC
HRAS G12D
Rel. mRNA
HRAS
NTC
BRAF V600E
Rel. mRNA
BRAF
NTC
PIK3CA
PIK3CA E545A
PIK3CA H1047R
Rel. mRNA
PIK3CA

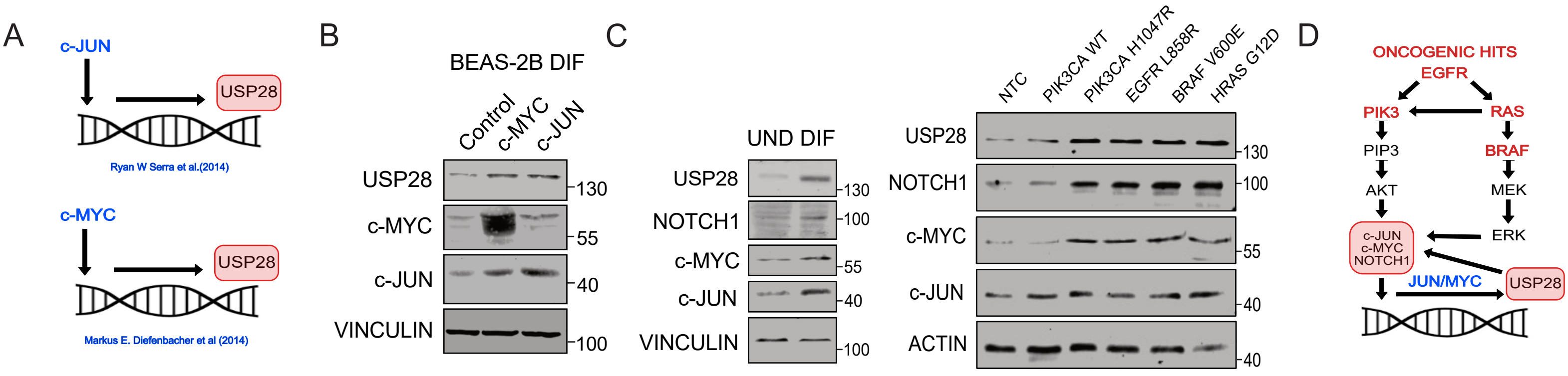

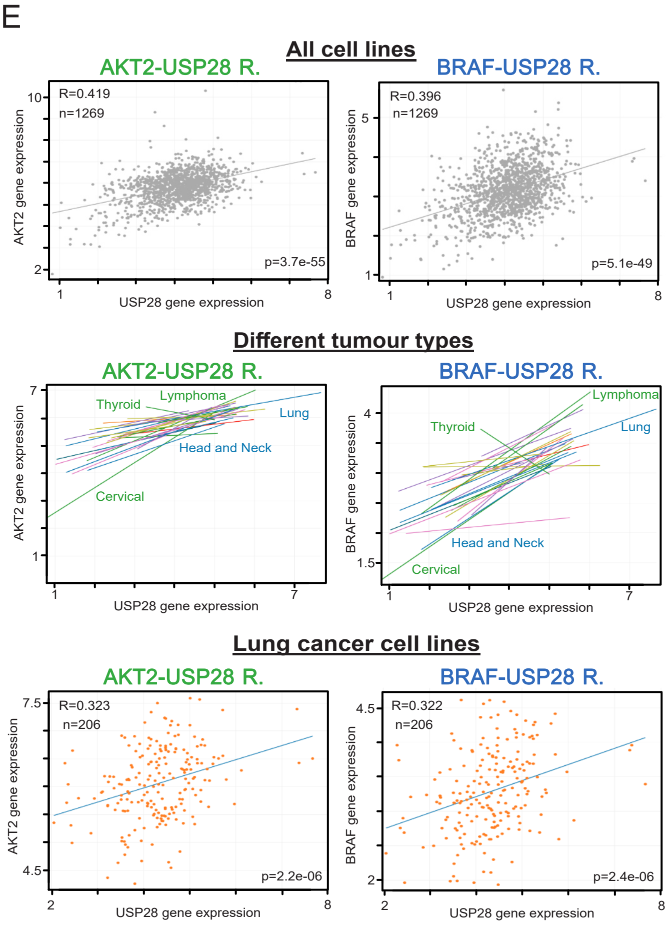

Cell line Tumour type	AKT2-USP28 R. Pearson R	AKT2-USP28 R. Slope	AKT2-USP28 R. p-value	BRAF-USP28 R. Pearson R	BRAF-USP28 R. Slope	BRAF-USP28 R. p-value
Thyroid Cancer	-0.253	-2.72E-1	4.28E-1	-0.665	-4.40E-1	1.82E-2
Skin Cancer	0.244	2.04E-1	4.34E-2	0.090	7.03E-2	4.62E-1
Sarcoma	0.579	4.16E-1	2.72E-4	0.386	2.60E-1	2.22E-2
Rhabdoid	0.569	8.10E-1	8.78E-3	0.467	6.02E-1	3.78E-2
Prostate Cancer	0.300	2.59E-1	5.13E-1	0.580	4.75E-1	1.72E-1
Pancreatic Cancer	0.427	5.44E-1	1.99E-3	0.422	2.68E-1	2.29E-3
Ovarian Cancer	0.321	3.50E-1	1.50E-2	0.569	4.00E-1	3.91E-6
Neuroblastoma	0.550	4.60E-1	3.59E-3	0.342	3.42E-1	8.74E-2
Myeloma	0.112	8.42E-2	5.56E-1	0.270	3.09E-1	1.48E-1
Lymphoma	0.685	6.06E-1	9.21E-12	0.602	4.84E-1	8.67E-9
Lung Cancer	0.323	2.54E-1	2.24E-6	0.322	2.34E-1	2.38E-6
Liver Cancer	0.068	4.60E-2	7.51E-1	0.110	8.65E-2	6.10E-1
Liposarcoma	0.818	5.94E-1	7.00E-3	0.218	6.46E-2	5.73E-1
Leukemia	0.436	3.09E-1	1.14E-5	0.378	2.45E-1	1.72E-4
Kidney Cancer	0.418	2.79E-1	1.55E-2	0.481	3.59E-1	4.61E-3
Head and Neck Cancer	0.580	4.91E-1	8.86E-5	0.573	4.40E-1	1.11E-4
Gastric Cancer	0.364	4.66E-1	1.95E-2	0.334	3.28E-1	3.30E-2
Gallbladder Cancer	0.834	4.60E-1	7.89E-2	0.480	1.71E-1	4.13E-1
Fibroblast	0.428	3.06E-1	6.60E-3	0.269	1.63E-1	9.75E-2
Eye Cancer	0.120	6.63E-2	7.98E-1	0.502	2.90E-1	2.51E-1
Esophageal Cancer	0.385	3.43E-1	2.98E-2	0.409	2.79E-1	2.02E-2
Endometrial/Uterine Cancer	0.303	3.99E-1	9.19E-2	0.672	5.28E-1	2.51E-5
Colon/Colorectal Cancer	0.575	4.52E-1	4.41E-7	0.521	3.37E-1	7.38E-6
Cervical Cancer	0.935	9.08E-1	8.25E-6	0.760	4.59E-1	4.12E-3
Breast Cancer	0.336	3.53E-1	1.15E-2	0.338	2.70E-1	1.08E-2
Brain Cancer	0.244	1.69E-1	3.12E-2	0.009	5.47E-3	9.35E-1
Bone Cancer	0.107	9.25E-2	5.36E-1	0.342	3.08E-1	4.09E-2
Bladder Cancer	0.191	1.72E-1	2.63E-1	0.212	1.66E-1	2.15E-1
Bile Duct Cancer	0.416	3.31E-1	1.78E-2	0.449	2.65E-1	9.92E-3

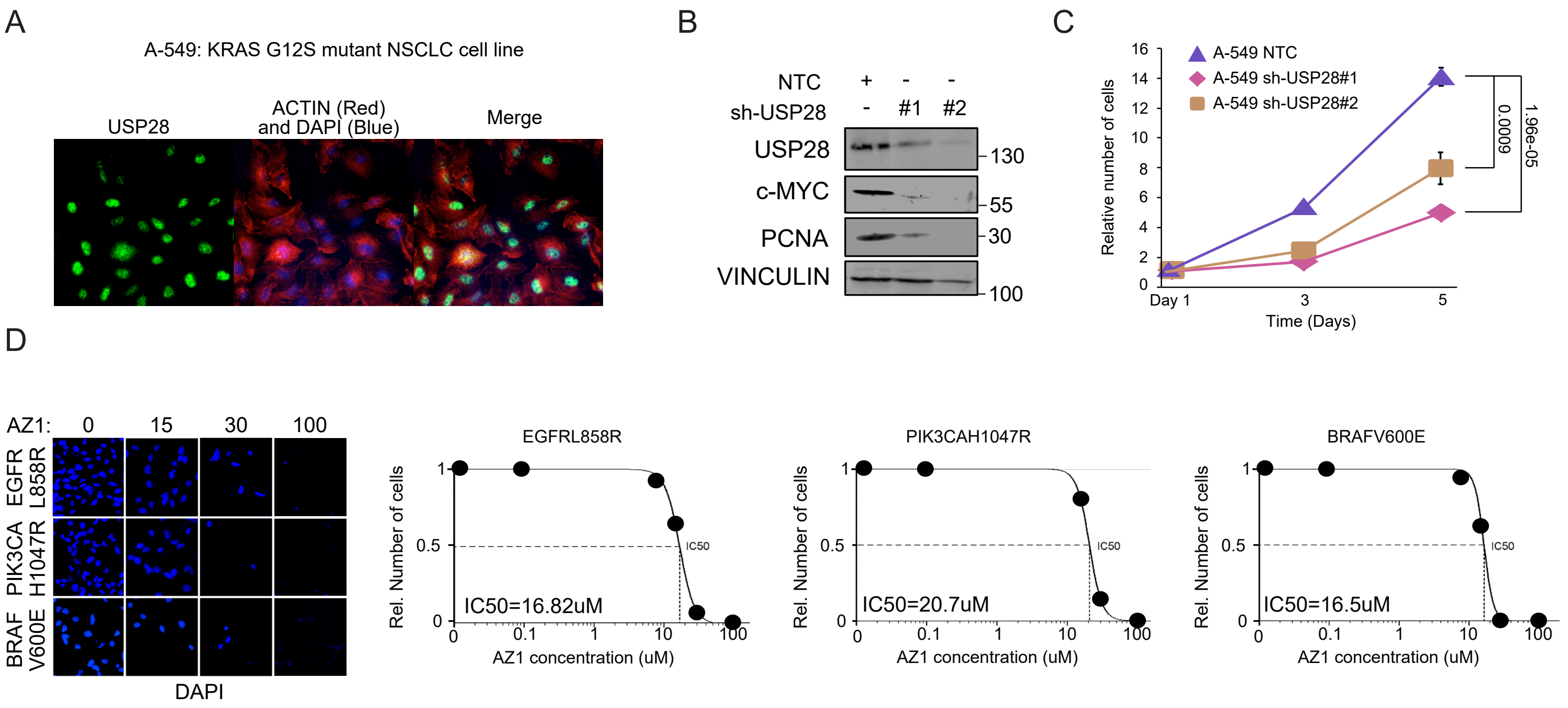

A
A-549: KRAS G12S mutant NSCLC cell line
USP28
ACTIN (Red) and DAPI (Blue)
Merge
B
NTC + - -
sh-USP28 - #1 #2
USP28
c-MYC
PCNA
VINCULIN
130
55
30
100
C
A-549 NTC
A-549 sh-USP28#1
A-549 sh-USP28#2
Relative number of cells
Time (Days)
Day 1
3
5
0.0009
1.96e-05
D
AZ1: 0 15 30 100
EGFR L858R
PIK3CA H1047R
BRAF V600E
DAPI
EGFRL858R
PIK3CAH1047R
BRAFV600E
Rel. Number of cells
AZ1 concentration (uM)
IC50
IC50=16.82uM
IC50=20.7uM
IC50=16.5uM

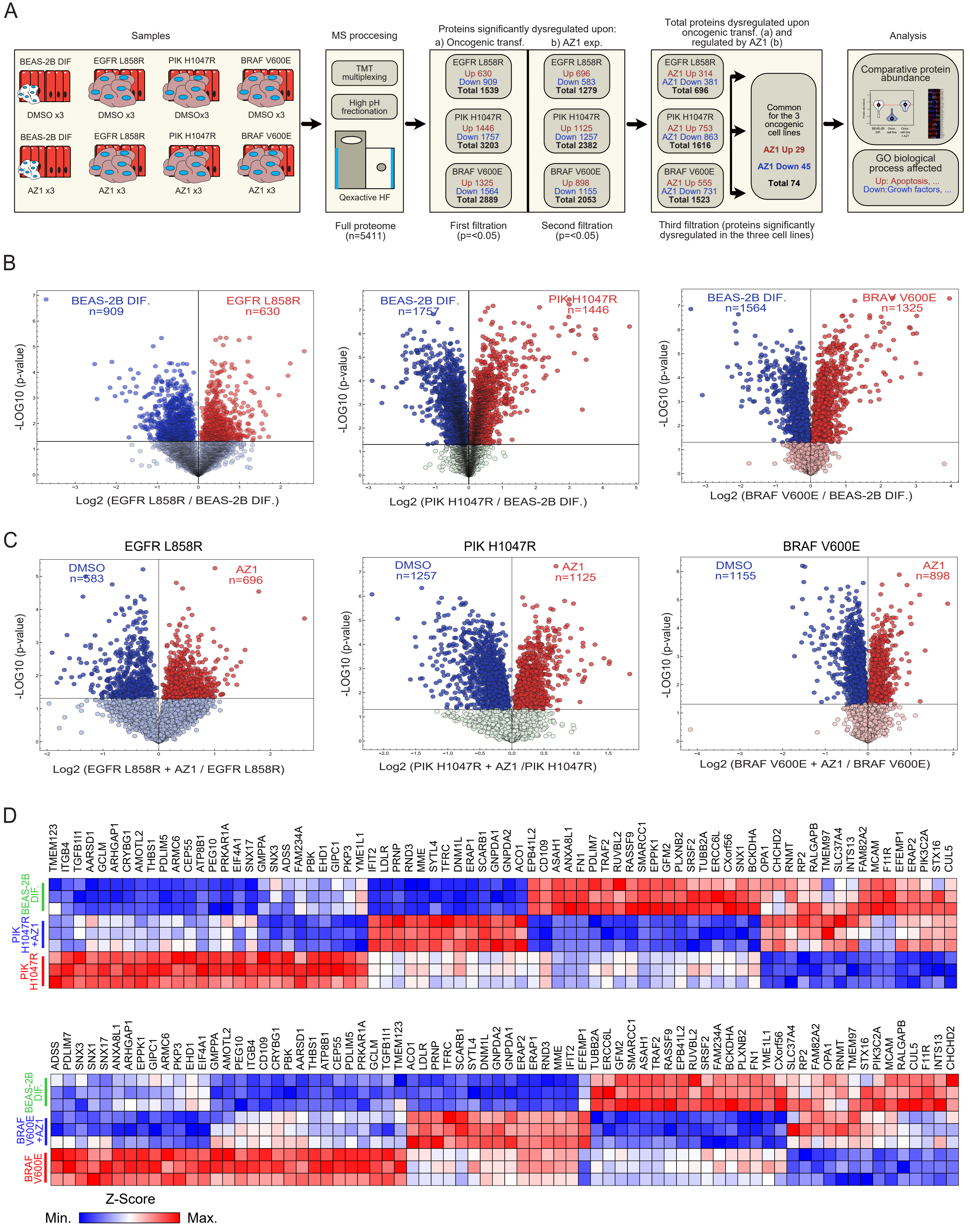
A
Samples
BEAS-2B DIF
EGFR L858R
PIK H1047R
BRAF V600E
DMSO x3
DMSO x3
DMSOx3
DMSO x3
BEAS-2B DIF
EGFR L858R
PIK H1047R
BRAF V600E
AZ1 x3
AZ1 x3
AZ1 x3
AZ1 x3
MS proccesing
TMT multiplexing
High pH frectionation
Qexactive HF
Full proteome (n=5411)
Proteins significantly dysregulated upon:
a) Oncogenic transf.
b) AZ1 exp.
EGFR L858R Up 630 Down 909 Total 1539
PIK H1047R Up 1446 Down 1757 Total 3203
BRAF V600E Up 1325 Down 1564 Total 2889
EGFR L858R Up 696 Down 583 Total 1279
PIK H1047R Up 1125 Down 1257 Total 2382
BRAF V600E Up 898 Down 1155 Total 2053
First filtration (p=<0.05)
Second filtration (p=<0.05)
Total proteins dysregulated upon oncogenic transf. (a) and regulated by AZ1 (b)
EGFR L858R AZ1 Up 314 AZ1 Down 381 Total 696
PIK H1047R AZ1 Up 753 AZ1 Down 863 Total 1616
BRAF V600E AZ1 Up 555 AZ1 Down 731 Total 1523
Common for the 3 oncogenic cell lines
AZ1 Up 29
AZ1 Down 45
Total 74
Third filtration (proteins significantly dysregulated in the three cell lines)
Analysis
Comparative protein abundance
GO biological process affected
Up: Apoptosis, ...
Down:Growth factors, ...
B
BEAS-2B DIF. n=909
EGFR L858R n=630
-LOG10 (p-value)
Log2 (EGFR L858R / BEAS-2B DIF.)
BEAS-2B DIF. n=1757
PIK H1047R n=1446
Log2 (PIK H1047R / BEAS-2B DIF.)
BEAS-2B DIF. n=1564
BRAF V600E n=1325
Log2 (BRAF V600E / BEAS-2B DIF.)
C
EGFR L858R
DMSO n=583
AZ1 n=696
Log2 (EGFR L858R + AZ1 / EGFR L858R)
PIK H1047R
DMSO n=1257
AZ1 n=1125
Log2 (PIK H1047R + AZ1 /PIK H1047R)
BRAF V600E
DMSO n=1155
AZ1 n=898
Log2 (BRAF V600E + AZ1 / BRAF V600E)
D
TMEM123 ITGB4 TGFB1I1 AARSD1 GCLM ARHGAP1 CRYBG1 AMOTL2 THBS1 PDLIM5 ARMC6 CEP55 ATP8B1 PEG10 PRKAR1A EIF4A1 SNX17 GMPPA SNX3 ADSS FAM234A PBK EHD1 GIPC1 PKP3 YME1L1 IFIT2 LDLR PRNP RND3 MME SYTL4 TFRC DNM1L ERAP1 SCARB1 GNPDA1 GNPDA2 ACO1 EPB41L2 CD109 ASAH1 ANXA8L1 FN1 PDLIM7 TRAF2 RUVBL2 RASSF9 SMARCC1 EPPK1 GFM2 PLXNB2 SRSF2 TUBB2A ERCC6L CXorf56 SNX1 BCKDHA OPA1 CHCHD2 RNMT RP2 RALGAPB TMEM97 SLC37A4 INTS13 FAM82A2 MCAM F11R EFEMP1 ERAP2 PIK3C2A STX16 CUL5
BEAS-2B DIF.
PIK H1047R +AZ1
PIK H1047R
ADSS PDLIM7 SNX3 SNX1 SNX17 ANXA8L1 ARHGAP1 EPPK1 GIPC1 ARMC6 PKP3 EHD1 EIF4A1 GMPPA AMOTL2 PEG10 CD109 ITGB4 CRYBG1 PBK AARSD1 THBS1 ATP8B1 CEP55 PDLIM5 PRKAR1A GCLM TGFB1I1 TMEM123 ACO1 LDLR PRNP TFRC SCARB1 SYTL4 DNM1L GNPDA2 GNPDA1 ERAP2 ERAP1 RND3 MME IFIT2 EFEMP1 TUBB2A ERCC6L GFM2 SMARCC1 ASAH1 TRAF2 RASSF9 EPB41L2 RUVBL2 SRSF2 FAM234A BCKDHA PLXNB2 FN1 YME1L1 CXorf56 SLC37A4 RP2 FAM82A2 OPA1 RNMT TMEM97 STX16 PIK3C2A MCAM RALGAPB CUL5 F11R INTS13 CHCHD2
BEAS-2B DIF.
BRAF V600E +AZ1
BRAF V600E
Z-Score
Min.
Max.

A

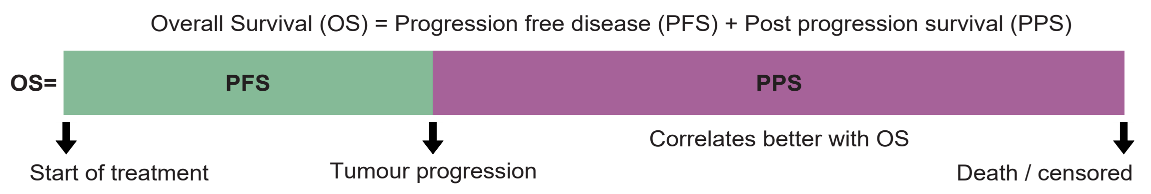

B

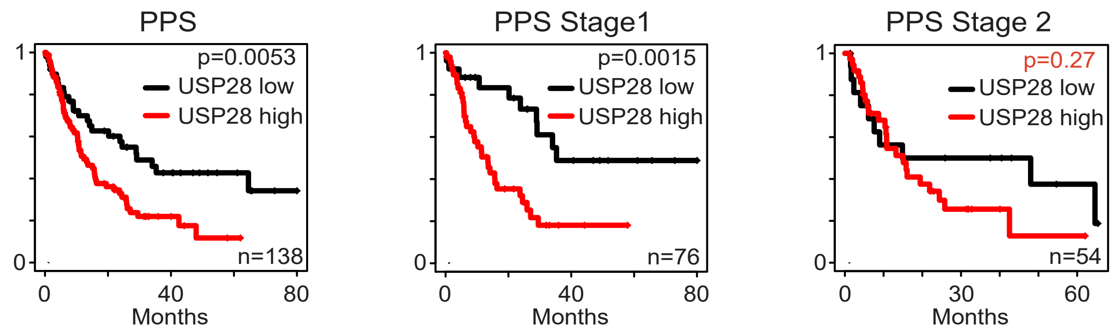

C

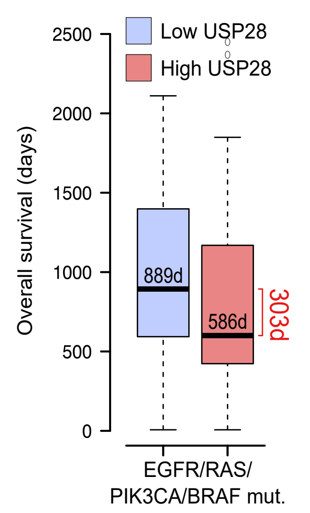

D

Median survival NSCLC patient (in days)

Mutation Status	EGFR		RAS		PIK3CA		BRAF		TP53	
	WT	Mutant	WT	Mutant	WT	Mutant	WT	Mutant	WT	Mutant
USP28 low	728	1147	925	728	728	882	745	1369	626	656
USP28 high	711	573	668	670	673	705	705	447	640	683
Different in days: (USP28 low-USP28 high)	17	**574**	257	58	55	177	40	**922**	-14	-27

n=1027

E

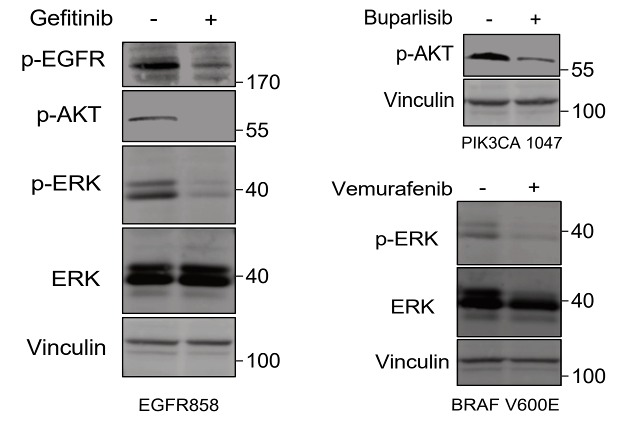

F

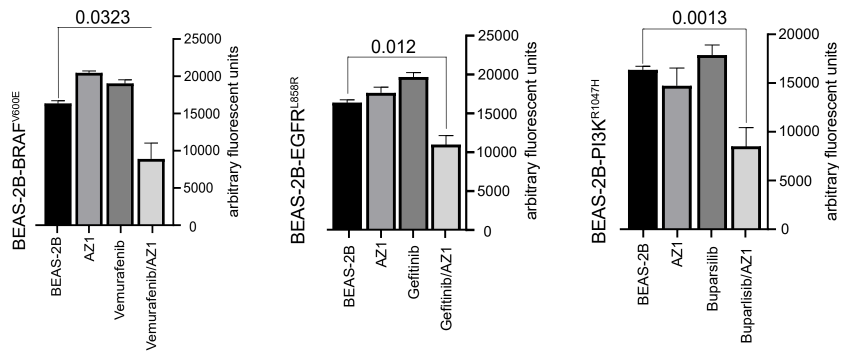

G

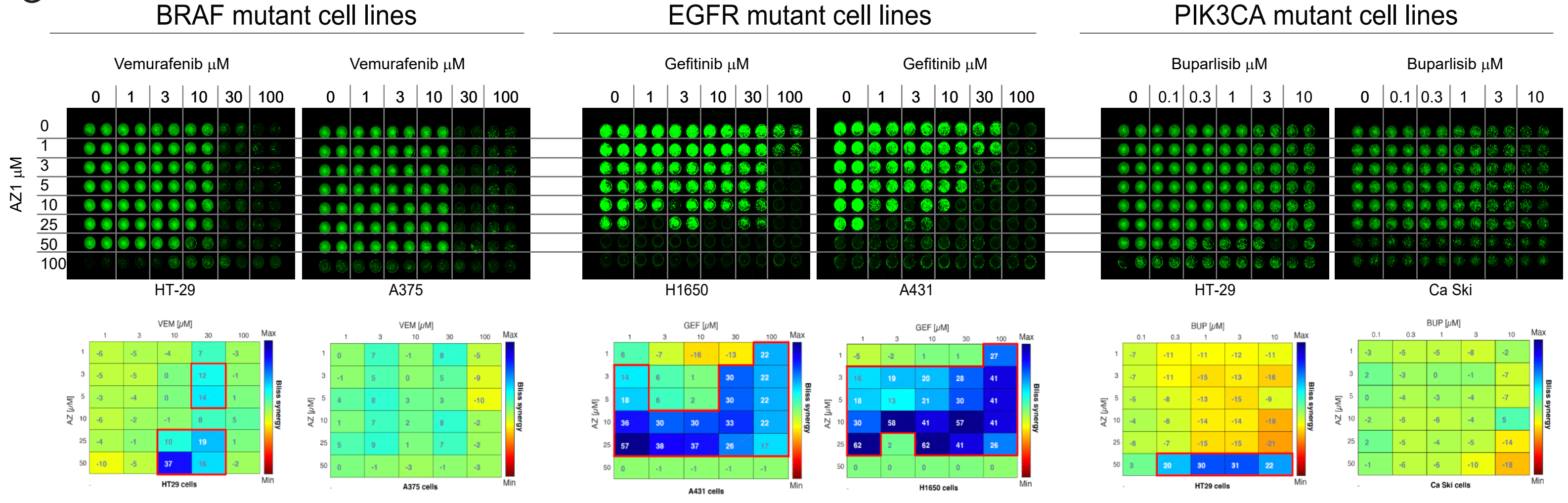

Supplement: Supplementary file 1 — Fig. S1. USP28 is upregulated irrespective of lung tumour subtype. Fig. S2. USP28 is required to establish oncogenic transformation in vivo. Fig. S3. USP28 is upregulated during early stages of transformation in the human cell line model system, BEAS‐2B. Fig. S4. Malignant transformation of BEAS‐2BDIF via EGFR‐PI3K‐MAP. Fig. S5. Oncogenic transformation via EGFR‐PI3K‐MAPK pathway upregulate USP28. Fig. S6. Malignant transformation renders tumour cells dependent on USP28. Fig. S7. Inhibition of USP28 via AZ1 ‘resets’ the proteome of oncogenic transduced cells towards a ‘nononcogenic’ state and induces proapoptotic signatures. Fig. S8. USP28 inhibition potentiates targeted molecular therapy. [file MOL2-16-3082-s002.pdf]
